# Quantum Chemical Microsolvation by Automated Water Placement

**DOI:** 10.3390/molecules26061793

**Published:** 2021-03-23

**Authors:** Miguel Steiner, Tanja Holzknecht, Michael Schauperl, Maren Podewitz

**Affiliations:** 1 Institute of General, Inorganic and Theoretical Chemistry, University of Innsbruck, Innrain 80-82, 6020 Innsbruck, Austria; miguel.steiner@phys.chem.ethz.ch (M.S.); tanjaholzknecht@gmail.com (T.H.); michael.schauperl@uibk.ac.at (M.S.); 2 Laboratorium für Physikalische Chemie, ETH Zürich, Vladimir-Prelog-Weg 2, 8093 Zürich, Switzerland

**Keywords:** microsolvation, implicit-explicit salvation, density functional theory, molecular dynamics, grid inhomogeneous solvation theory, bulk phase

## Abstract

We developed a quantitative approach to quantum chemical microsolvation. Key in our methodology is the automatic placement of individual solvent molecules based on the free energy solvation thermodynamics derived from molecular dynamics (MD) simulations and grid inhomogeneous solvation theory (GIST). This protocol enabled us to rigorously define the number, position, and orientation of individual solvent molecules and to determine their interaction with the solute based on physical quantities. The generated solute–solvent clusters served as an input for subsequent quantum chemical investigations. We showcased the applicability, scope, and limitations of this computational approach for a number of small molecules, including urea, 2-aminobenzothiazole, (+)-*syn*-benzotriborneol, benzoic acid, and helicene. Our results show excellent agreement with the available *ab initio* molecular dynamics data and experimental results.

## 1. Introduction

Solvents play a pivotal role in solution phase chemistry. The efficiency of many chemical transformations ranging from organic reactions to (transition-)metal catalyzed processes is intricately linked to the solvent in which they are carried out [1,2,3,4]. As a result, the chemically relevant solution phase conformations of the reactants, intermediates, and products may differ significantly from their gas phase or solid-state counterparts and can even depend on the specific solvent [5]. This is in particular true for bio- or biomimetic molecules and biochemical reactions that take place in water.

To accurately account for the reactivity in the condensed phase, a reliable characterization is necessary. Despite experimental advances, the achieved temporal and spatial resolution is often not sufficient to decipher the impact of complex solvent effects. Theoretical investigations are an attractive alternative to fill this gap, but the accurate treatment of (explicit) solvation is still an active field of research in computational chemistry [5,6,7,8].

Yet, several strategies exist. In the simplest model, only bulk properties of the solvent are considered. These so-called implicit solvation models are computationally cost-effective and have long been applied in computational chemistry [9,10,11,12]. However, they fail if explicit solute–solvent interactions are expected to play a role. At the other end of the spectrum, condensed phase approaches exist, which take a large number of solvent molecules into account. Most accurately, solute and solvent are treated quantum-mechanically. However, such *ab initio* molecular dynamics (MD) approaches are quite expensive and limited to relatively small systems [13,14,15]. As a third strategy, explicit–implicit solvation approaches—also referred to as microsolvation—have emerged as a low-cost alternative. Here, a small number of explicit solvent molecules are incorporated in a quantum chemical calculation [8,16,17,18,19,20].

However, microsolvation approaches implicate new challenges such as how many solvent molecules to include, where to put them, and how to orient them. Until today, numerous methodologies to microsolvation have been reported, which can be largely divided into two categories: bottom-up approaches, where starting from the solute alone, explicit solvent molecules are successively added, and top-down approaches, where starting from a condensed phase calculation—often a classical MD simulation—a few explicit solvent molecules are selected.

Bottom-up approaches comprise, for example, the cluster-continuum approach by Pliego and coworkers, where individual solvent molecules are added and the free energy of solvation, Δ*G*_solv_, is calculated quantum-mechanically. The number of solvents that minimizes Δ*G*_solv_ is then taken for further investigations. Alternative approaches are based on an analysis of the molecular electrostatic potential to identify interaction sites [21,22,23]. Following a related idea, Sure et al. proposed a protocol based on the analysis of the screening charge densities [24]. Another idea is to start from “idealized” solvent geometries, such as the structures of clathrates, to describe microsolvated amino acids [25]. Simm and coworkers developed a stochastic approach, where interaction surfaces of solute and solvent are identified and randomly combined. Additional conformational sampling ensures generation of a structural ensemble [26]. More recently, global optimization procedures in combination with machine learning techniques have emerged to speed up the generation and identification of the most stable microsolvated clusters [27,28].

However, a general problem arises when taking the solute as starting point to build the microsolvation model: the question is how to define a reliable solute conformation without knowing it a priori. Convergence may be challenged if the structure changes drastically with increasing number of explicitly treated solvent molecules. In addition, evaluation of many solute–solvent cluster configurations may be necessary to identify the most stable arrangement, which is computationally demanding.

Top-down approaches circumvent this issue by starting the investigation from a condensed phase structure. Here, microsolvated solute structures are often derived from snapshots of a classical MD simulation or from Monte Carlo sampling. Typically, all solvent molecules within a certain distance to the solute are included in a follow-up quantum chemical calculation (for selected examples, see Refs. [17,29,30,31]). Following a slightly different approach, Nocito and Baran developed an averaged condensed phase environment derived from snapshots of classical MD trajectories to be applied in quantum chemical calculations [32].

Several studies showed the practicality of such approaches to microsolvation. For example, solute–solvent cluster models were found to improve the description of electronic excitation spectra, [29,32,33] or of IR and Raman spectra [30]. However, in particular in top-down approaches, the number of explicit solvent molecules to be included in the cluster model is often justified based on the distance to the solute alone. A rigorous criterion to select the number of explicit solvent molecules to be included in the microsolvation model based on physical properties is missing. This can be attributed to the inherent difficulty in condensed phase (classical) MD simulations to define the solute–solvent interaction strength and to assign locations to the solvent molecules [34].

However, in biomolecular simulations, the quantification of solute–solvent interactions and the identification of hydration sites—that is, the location of water molecules that show slow or no movement during the simulation—are an important goal due to its relevance in drug design [35,36,37,38,39,40]. Several computational tools exist to aid this procedure [37,39,40,41]. For example, grid inhomogeneous solvation theory (GIST) allows the analysis and thermodynamic quantification of the solute–solvent interaction on a grid over the whole trajectory [42,43]. Prior studies have shown that GIST can be used to identify favorable interaction sites in heterocycles [44,45] or in biomolecules, [43,46,47] for example, in antifreeze proteins [47] and kinases [46]. In the latter studies, the grid points were further analyzed to identify positions with high water density to visualize locations of strongly interacting solvent molecules. Yet, the only purpose of these initial studies was to enhance visualization, and the analysis determined oxygen positions only. 

In the present study, we build on this idea to propose a rigorous protocol to quantum chemical microsolvation by automated water placement based on physical properties. We combined the GIST analysis with an evaluation of the averaged solvent density to place individual water molecules and to quantify their interaction with the solute. A crucial point was the development of an algorithm to assign hydrogen positions to obtain water orientations. Our approach is implemented in a fully automated Python script. The user can choose the number of explicit solvent molecules for subsequent quantum chemical studies based on their position and interaction strength. We chose five different small molecules, which differ in size and polarity, to test our methodology: urea and benzoic acid due to their biological relevance; (+)-*syn*-benzotriborneol because of its ability to act as a water receptor [48]; helicene because of its apolar nature; and 2-aminobenzothiazole, which can undergo water-assisted tautomerization [49]. All of these molecules have previously been investigated either experimentally by rotational spectroscopy as well as X-ray and neutron diffraction analysis or theoretically by quantum chemical or molecular dynamics studies [48,49,50,51,52,53,54,55,56,57,58,59].

After a brief introduction to GIST and to our methodology, the capabilities of our top-down approach to quantum chemical microsolvation are illustrated in the example of the previously introduced five small molecules. Thereby, we aimed at including a variety of compounds that differ in size and polarity. 

## 2. Grid Inhomogeneous Solvation Theory (GIST)

Grid inhomogeneous solvation theory (GIST) is a method to calculate the free energy of solvation for a fixed solute conformation by evaluating (local) thermodynamics—that is, solute–solvent interactions—on a grid [42,43,46,47]. While a detailed derivation of the GIST formalism can be found elsewhere, [42,43] we briefly introduce the theoretical foundations here.

In GIST, the equations of inhomogeneous solvation theory (IST) [60] are solved numerically by sampling a constrained solute in solution via MD simulations and assigning solvent molecules to a three-dimensional grid. The result of this analysis is a three-dimensional density map of the solvent around the solute. From this map, the free energy of solvation Δ*A*_solv_ can be calculated as a sum over the individual contributions of all grid voxels *r* according to Equation (1).

(1) $$\Delta A_{\mathrm{solv}}=\underset{r}{\sum }\Delta A_{r}$$

The free energy of solvation of each grid voxel *r* is split into an energy and an entropy term as described in Equation (2).

(2) $$\Delta A_{r}=\Delta E_{r}-T\Delta S_{r}$$

The energy term is further split into solute–solvent (Δ*E*_sw_) and solvent–solvent (Δ*E*_ww_) interactions (see Equation (3)).

(3) $$\Delta E_{r}=\Delta E_{\mathrm{sw}}+\Delta E_{\mathrm{ww}}$$

As our analysis is limited to water only, we refer to the solvent by the subscript “w.” The entropy is split into a translational and an orientational term (Equations (4) and (5)), which are calculated as a Shannon entropy approximated by a nearest-neighbor approach,

(4) $$S_{r}^{\mathrm{trans}}=R\left(\gamma +\frac{1}{N_{r}}\overset{N_{r}}{\underset{i=1}{\sum }}\ln\left(\frac{N_{f}\rho^{0}4\pi \cdot d_{\mathrm{trans}}^{3}}{3}\right)\right)$$

(5) $$S_{r}^{\mathrm{orient}}=R\left(\gamma +\frac{1}{N_{r}}\overset{N_{r}}{\underset{i=1}{\sum }}\ln\left(\frac{N_{f}{\left({\Delta \omega }_{i}\right)}^{3}}{6\pi }\right)\right)$$

with *R* being the ideal gas constant, $N_{r}$ the total number of solvent molecules in voxel r during the simulation, and γ Euler’s constant, which corrects for an asymptotic bias. $N_{f}$ is the number of frames of the simulation, $\rho^{0}$ is the reference density of the solvent model, $d_{\mathrm{trans}}$ is the closest distance and Δω is the closest angular distance between two solvent molecules.

By quantifying the individual enthalpic and entropic contributions to the free energy of solvation, GIST allows us to identify interaction sites, or rather grid voxels, which show strong interactions with the solute. Identification of such sites is essential for the location of solvent molecules in a microsolvation approach.

## 3. Computational Methodology

### 3.1. Computational Protocol to Microsolvation

**Figure 1:**
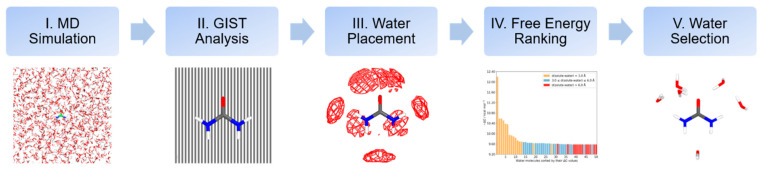
Workflow for the automated generation of microsolvated structures retrieved with our Free Energy Based Identification of Solvation Sites (FEBISS) algorithm from molecular dynamics (MD) data and grid inhomogeneous solvation theory (GIST) analysis.

Our computational protocol to quantum chemical microsolvation is shown in Figure 1. It started with sampling the solute in solution by molecular dynamics (MD) simulations (I). Second, we performed a grid inhomogeneous solvation theory (GIST) analysis of the obtained trajectory (II) to identify favorable interaction sites with the solute. Based on the interaction sites and the water density of the trajectory, individual water molecules were placed and oriented (III) and then ranked according to their interaction strength with the solute (IV). As a last step, the user could select the number of water molecules for subsequent optimizations (V).

The workflow after the simulation was automated and autonomously handled by our program Free Energy Based Identification of Solvation Sites (FEBISS).

MD Simulations and GIST Analysis. Prior to any analysis, an MD simulation of the solute in the solvent (here, in water) had to be performed. In case the solute showed significant dynamics, a clustering of the trajectory had to be performed to identify the most populated conformations. The most stable solute conformation or the cluster representatives were then simulated with the solute coordinates being constrained as required for GIST. The MD simulations can be performed with any software (any force field and water model) as long as the written topology and trajectory files can be read by the freely available open-source software Cpptraj [61].

The obtained trajectory was then analyzed with GIST to calculate the thermodynamic contributions of the solute–solvent interaction on a grid (with a spacing of 0.5 Å). As outlined above, GIST discretizes the trajectory and allows to identify solute–solvent interaction sites (grid voxels) with specific solvation thermodynamics. For the purpose of our computational protocol to microsolvation, we were solely interested in interaction sites (grid voxels) that showed a favorable (negative) free energy of solvation Δ*A_r_* and a high water density.

Water Placement. In our protocol, solvent positions were assigned based on the averaged solvent density around the solute and the discretized free energies of solvation. First, the algorithm identified the grid point of highest water density. A water molecule was placed at this position and the density of one water molecule was subtracted from this grid point. If the density at this grid point was lower than that of a single water molecule, neighboring voxels were included until the sum of their densities suffices. The reference density of one water molecule was taken from the applied water model, here TIP3P [62]. The assignment procedure was repeated until 95% of the total water density was allocated to individual water atoms. Second, the free energy of the selected grid voxel was density-weighted and assigned to the positioned water molecule. This ensured a more accurate representation of the free energy of solvation in the case of insufficient sampling. The water molecules were assigned the free energy value from the grid voxel, where the oxygen nucleus was placed.

**Figure 2:**
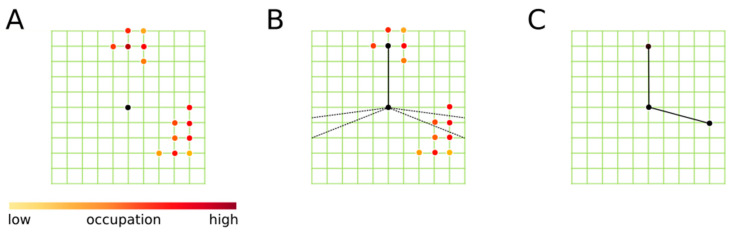
Schematic representation of the hydrogen-positioning algorithm. (**A**) The black center dot represents the oxygen atom, whereas the color-coded dots represent the occupation number of the protons on the grid. (**B**) Placement of the first proton at the highest grid occupation. To obtain a reasonable water geometry with an H–O–H angle of 104.5 ± 5.0°, the position of the second proton is limited to the space in between the dotted lines, which corresponds to a spherical segment in three dimensions. (**C**) Resulting water orientation.

By this procedure, water molecules at points of high density and favorable free energy could be identified. Next, positions of the protons were retrieved by an algorithm sketched in Figure 2. Here, the positions of all protons were analyzed for each frame of the simulation. These proton positions were binned onto a smaller grid with a spacing of 0.1 Å, as shown in Figure 2A. The first proton was then placed on the voxel with the highest occupation number. For the second proton, our algorithm only considered positions resulting in a water angle that deviated from the equilibrium angle by less than 5°, as shown in Figure 2B. Within this spherical segment, the second proton was placed at the voxel with the highest occupation number (Figure 2C). The specific value of the H–O–H angle depends on the applied water model in the simulation. For our study, we used the TIP3P water model, [62] with an equilibrium H–O–H angle of 104.5°.

Free Energy Ranking. We assigned each placed water molecule the free energy value Δ*A* of the voxel where the oxygen was placed, weighted by the density of that voxel. The water molecules were then sorted by their Δ*A* values and displayed as a bar chart, where the height denotes their free energy and a color coding indicates their distance to the solute. We may categorize them into three groups: (i) water molecules with a distance < 3 Å, color-coded in orange: such water molecules typically describe the first solvation shell in hydrophilic regions of the solute; (ii) water molecules with distances between 3 Å < *d* < 6 Å, color-coded in blue: such molecules comprise either the second solvation shell in hydrophilic regions of the solute or the first solvation shell in hydrophobic regions; and (iii) water molecules with *d* > 6 Å, depicted in red: they are typical “bulk-like” molecules with almost no interaction with the solute.

Water Selection within the Graphical User Interface (GUI). A bar chart displays the ranking of the placed water molecules and serves as a graphical user interface (GUI). Users can select the water molecules for the microsolvated structure based on the interaction energy (solvation free energy) and the distance to the solute by clicking on the individual bars. Complementary radial distribution functions (RDF) plots of the simulation including integral values can further aid the user in the selection of the number of solvent molecules.

Implementation and Usage. To obtain microsolvated solute structures with our computational protocol, any MD simulation package can be used as long as it generates a trajectory and topology file that can be converted to be readable by the freely available open-source software Cpptraj [61]. Most conveniently, this can be achieved with the Amber package [63,64].

Besides the MD simulation, our Python program FEBISS handles the entire workflow as outlined above. It calls the GIST analysis and performs the newly implemented water placement—both steps are integrated in the C++ open-source software Cpptraj [61]. It generates the interactive bar charts so that the user can select the number of water molecules; it saves the solvated structures as pdb files and automatically displays them in PyMOL [65].

FEBISS is available on Github [66] and via pip for Python3 free of charge. To increase performance, our protocol makes use of the recently published GPU-accelerated GIST version, [67] while patch are files available on Github [68]. Our algorithm is directly implemented within the GIST analysis and can be invoked with the additional keyword *febiss*. Our Python program is able to install all dependencies automatically and performs the GIST analysis and water placement by calling Cpptraj. The analysis and the interactive bar chart can also be customized with an easy-to-use yaml input file.

### 3.2. Molecular Dynamics Simulation Details

All structures were generated with the Gaussian 16 program, [69] with the exception of (+)-*syn*-benzotriborneol, which was retrieved from the available crystal structure (CDCC code: 690687) [48]. The simulation setup, including the parameterization of the systems, as well as the final production runs were performed using the Amber18 simulation package [63,64]. For the parametrization, restrained electrostatic potential (RESP) charges [70] were calculated with HF/6-31G* for each system, using the Gaussian 16 program [69]. For the sake of comparison AM1-BCC charges [71] were also calculated for urea. This step was followed by the assignment of the atom types to all structures, using the generalized Amber force field (GAFF) [72] and the antechamber module of AMBERTools19 [64]. The systems were then solvated by applying an octahedral TIP3P [62] water box, maintaining a minimum distance of 20 Å between the solute and the edges of the box. The solvated systems were subjected to an equilibration run, following a thorough protocol by Wallnöfer et al. [73]. The final production runs were carried out in NVT ensembles, where the number of particles N, the volume V and the temperature T are kept constant with T = 300 K, using the GPU implementation of the pmemd module of AMBER 18, [64,74] whereby the temperature was controlled by applying the Langevin thermostat [75]. To achieve a higher performance, periodic boundary conditions and the smooth particle–mesh Ewald approach [76] were applied in all simulations. Additionally, the SHAKE algorithm [77] was used to constrain bonds involving hydrogen atoms. Regarding the restrained NVT runs for the GIST analysis, the whole solute was restrained with a force constant of 100 kcal/mol Å^2^. The time step was set to 2 fs, and snapshots of the systems coordinates were saved every 10 ps. The final trajectories comprised 30,000 frames, which corresponds to 300 ns of NVT MD simulation time. 

### 3.3. Data Analysis and Reference Settings

For the GIST analysis, we applied a 30 Å × 30 Å × 30 Å cubic grid with a spacing of 0.5 Å to obtain a reasonable balance between accuracy and convergence [43]. As all of the thermodynamic terms are state functions, reference values corresponding to the pure solvent state were needed. Throughout this paper, the reference solvent–solvent interaction was set to *E*_ww_ = −9.533 kcal mol^−1^ (taken from the TIP3P model), [62] whereas *E*_sw_, *S*_trans_, and *S*_rot_ were all set to zero. 

All data analysis was handled by our program FEBISS, which calls the Cpptraj program [61] from the Amber tools package [64]. For the sake of comparison, we applied the 3-dimensional reference interaction site model (3D-RISM), [78] using the Solvent Analysis tool implemented in the MOE software [79]. To visualize the calculated 3D-RISM water densities, the Solvent Analysis Grid panel was used. We set a minimum distance of 15 Å between the solute and the edges of the solvent box and defined a tight convergence criterion. The remaining parameters were kept to their default values. We selected a cut-off of 2.9 Å and 1.9 Å for the RDFs of the water oxygen and hydrogen atoms, respectively, which aligns with the maximum RDFs determined in previously published results [50,52].

### 3.4. Quantum Chemical Calculations

Initial microsolvated structures retrieved from the FEBISS analysis were subjected to an optimization using density functional theory (DFT). All calculations were performed using the Turbomole electronic structure package (Version: 7.3) [80,81]. To comply with the already published data, all systems were optimized with the hybrid density functional B3LYP [82,83,84] and the triple-zeta basis set def2-TZVP [85]. Dispersion contributions in the DFT calculations were invoked by empirical atom-pairwise dispersion corrections with the Becke–Johnson damping [86,87]. In addition to the explicitly described water molecules, bulk solvent effects were treated implicitly, using the conductor-like screening model (COSMO) [88,89] as implemented in Turbomole. A dielectric constant of *ε* = 78.5 was predefined for all calculations. In this approach, the solute is placed in a cavity of a dielectric continuum. The cavity is defined by the solvent accessible surface obtained by applying increased van der Waals radii around each atom. In the case of explicit–implicit solvation models, this is done for each explicitly described molecule (solute and solvents). It has to be ensured that the cavity is meaningful and closed, and this was the case in all our calculations. Analysis of the Hessian verified that all optimized structures only had positive eigenvalues and were indeed energy minima.

The optimized geometries of the microsolvated clusters were depicted with the molecular visualization program PyMOL [65].

## 4. Results

**Scheme 1:**
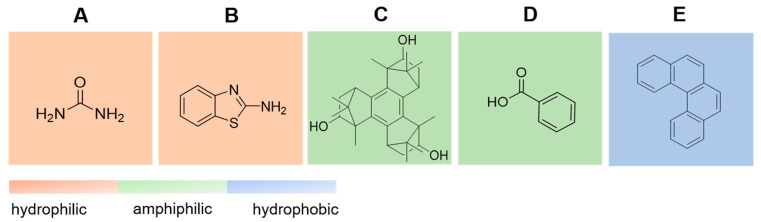
Lewis formulae of the molecules included in our test set. The structures are ordered according to their polarity, starting with the most hydrophilic and ending with the most hydrophobic system: urea (**A**), 2-aminobenzothiazole (**B**), (+)-*syn*-benzotriborneol (**C**), benzoic acid (**D**), and helicene (**E**).

We tested the applicability and accuracy of our automated solvent placement protocol on the following five systems: urea, 2-aminobenzothiazole, (+)-*syn*-benzotriborneol, benzoic acid, and helicene (see Scheme 1). The results of each investigated compound are discussed in order of decreasing polarity.

### 4.1. Urea–Water Complexes

**Figure 3:**
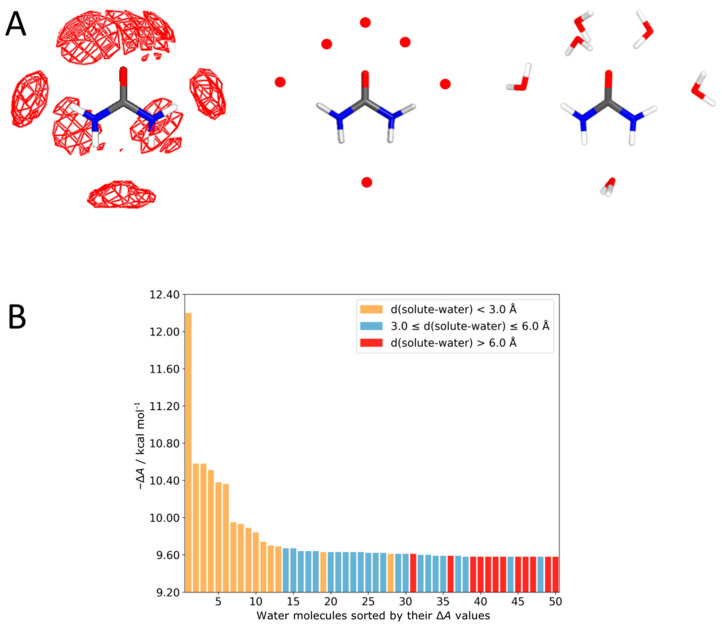
(**A**) Representation of the automated water placement algorithm. Left: Urea’s water density map. Middle: Ascertained water oxygen positions. Right: Final solvated complex with appropriate water hydrogen orientations. (**B**) Ranking of the automatically placed water molecules around the urea molecule. The free energy of the first 50 water molecules is depicted. The distances between solute and water are color-coded, whereby orange denotes a solute–solvent distance of less than 3 Å; blue, a solute–water distance between 3 and 6 Å; and red, a solute–water distance of more than 6 Å.

We started the analysis with the urea molecule, which is the smallest compound of our test library. A 300 ns restrained NVT simulation was performed and the trajectory was subjected to a GIST analysis. The main steps of the FEBISS algorithm are depicted in Figure 3A: This process includes the identification of areas with high water density (Figure 3A (left)), the placement of water oxygen atoms (Figure 3A (middle)), and the assignment of favorable water hydrogen orientations (Figure 3A (right)). At the end of the FEBISS calculation, thermodynamic parameters for all water molecules were retrieved and the solvent molecules were ranked according to their free energy values. The resulting bar chart is depicted in Figure 3B. This graph allows the identification of thermodynamically favorable water molecules and, at the same time, permits the selection of water molecules with a predefined solute–water distance cut-off. The FEBISS graph of the urea system depicts 13 consecutive water molecules with a solute–water distance below 3 Å (Figure 3B, orange bars). Six of these water molecules have a Δ*A* value lower than −10.00 kcal/mol. Additionally, we can point out one strongly bound water molecule with a Δ*A* value of −12.20 kcal/mol. 

**Figure 4:**
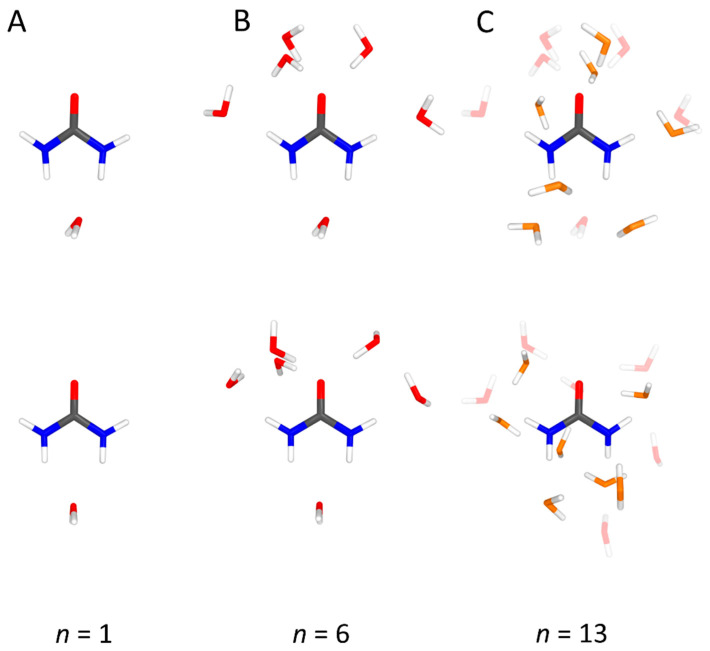
Representation of the urea–(H_2_O)*_n_* complexes before (upper row) and after (lower row) quantum chemical optimization with B3LYP/def2-TZVP/D3 in implicit solvent, with *n* = 1 (**A**), *n* = 6 (**B**), and *n* = 13 (**C**) water molecules. The six water molecules with the lowest Δ*A* value are colored in red, while the additional seven water molecules in the urea–(H_2_O)_13_ complex are colored in orange.

Based on the FEBISS bar chart, we selected three urea–(H_2_O)*_n_* complexes, with *n* corresponding to 1, 6, and 13 water molecules. Both the urea–(H_2_O)*_n_* complexes directly retrieved from the FEBISS calculation and the urea–(H_2_O)*_n_* complexes after DFT optimization with the COSMO solvation model are represented in Figure 4. The lowest Δ*A* value (FEBISS graph in Figure 3) matches a single water molecule located in between the two urea amide groups (Figure 4A and Figure S2), with Δ*A* = − 12.20 kcal/mol. The interaction is characterized by a bifurcated hydrogen bond, formed by the water oxygen atom and the amide hydrogen atoms of urea. Regarding the urea–(H_2_O)_6_ complex (Figure 4B), we can observe three additional water molecules coordinating urea’s carbonyl oxygen in an elliptical configuration with Δ*A* = −10.50 kcal/mol (compare also Figure S2). All three water molecules interact directly with the urea oxygen atom, whereby the average distance for the O_urea_–H_water_ atom pair is 1.87 Å and 1.79 Å in the non-optimized and optimized water cluster, respectively. Furthermore, we can observe two more water molecules in the region that encompasses the urea oxygen atom and the amide groups. Concerning the urea–(H_2_O)_13_ complex (Figure 4C), we can see that seven additional water molecules are distributed around the urea molecule; these do not directly interact with the solute, but form hydrogen bonds with the already placed water molecules.

It is noteworthy that the FEBISS chart and the water positioning looked different when AM1-BCC [71] rather than RESP [70] charges were used (Figure S3). With AM1-BCC charges, four water molecules rather than three were found to be coordinated to the oxygen and one was localized between the amide groups.

When comparing radial distribution functions (RDFs) from our simulation to previously published *ab initio* molecular dynamics data, [52] we see a good agreement (see Figure S1 in Supplementary Materials). This fact proved that the sampling time of our simulation was sufficient, and that classical MD simulations are justified as input for our water placement algorithm FEBISS.

**Figure 5:**
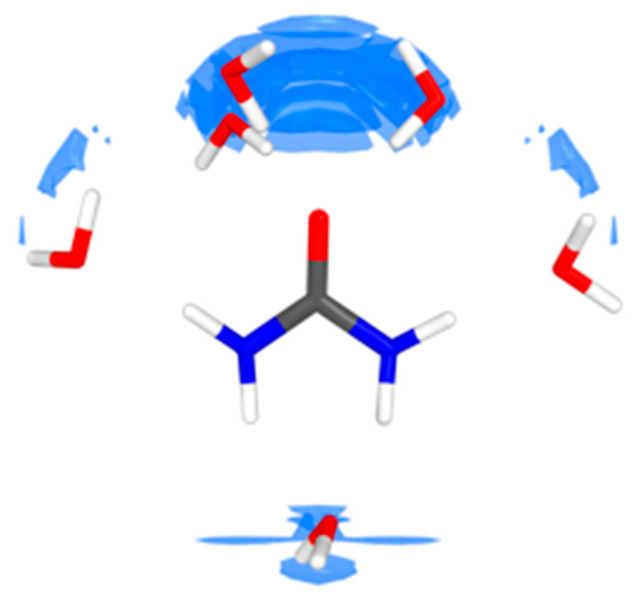
Comparison of the water placements around urea obtained with the FEBISS algorithm and the 3D reference interaction site model (3D-RISM) approach. The urea–(H_2_O)_6_ complex is represented with sticks, while the water densities obtained with the 3D-RISM approach are shaded in blue.

For further validation, we compared our results with the urea–water interaction sites determined by the 3D reference interaction site model (3D-RISM). In Figure 5, an overlay of the optimized urea–(H_2_O)_6_ complex and the solvation sites obtained with the 3D-RISM methodology is shown. We find a very good agreement between the water positions obtained with our FEBISS algorithm and the high-density water locations determined with the 3D-RISM calculation. Both methodologies agree on the existence of a water molecule in between the two amide groups, as well as on water molecules surrounding the urea oxygen atom in a hemispherical manner. However, with our FEBISS algorithm, we were able to identify two more explicit water positions, which span the region between the urea oxygen and urea amide groups, which are less recognizable in the 3D-RISM results.

In addition, we investigated the dipole moments of the urea–water clusters. While urea in implicit solvent has a dipole moment of 6.10 D, the addition of one water molecule increased this value to 9.24 D, whereas for the urea–(H_2_O)_6_ cluster, a dipole moment of 9.57 D was found. On further increasing the number of explicitly treated waters to 13, the dipole moment decreased to 2.98 D.

### 4.2. Aminobenzothiazole–Water Complexes

**Figure 6:**
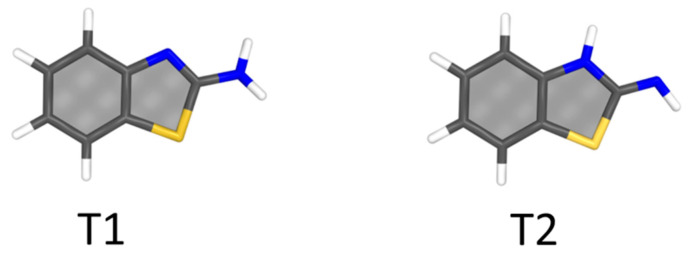
Quantum-chemically predicted conformations of T1 and T2. The relative electronic energy difference Δ*E*_rel_ *= E*(T2) − *E*(T1) is 5.4 kcal/mol in the gas phase and 5.6 kcal/mol in the implicit solvent when calculated with B3LYP/def2-TZVP/D3.

2-Aminobenzothiazole (ABT) served as another test system in our study (see Scheme 1). It exists in two tautomer conformations, the amino (T1) and the trans-imino (T2) structural isomers, depicted in Figure 6. Previous studies have shown that water assists the tautomerization process and the conversion from T1 to T2. A microsolvation model was required to calculate meaningful transition state structures and reaction barriers, while the number and location of the explicitly treated water molecules was determined by trial and error [49]. Our goal was to investigate whether our microsolvation protocol is able to yield reasonable microsolvated structures. Therefore, we analyzed both tautomeric forms with our FEBISS algorithm and assessed the number, location, and orientation of individual solvent molecules as well as their impact on the proton transfer. 

Initial DFT structure optimizations in the implicit solvent confirmed the amino isomer (T1) to be more stable—by 5.4 kcal/mol in the gas phase and by 5.6 kcal/mol in the implicit solvent.

**Figure 7:**
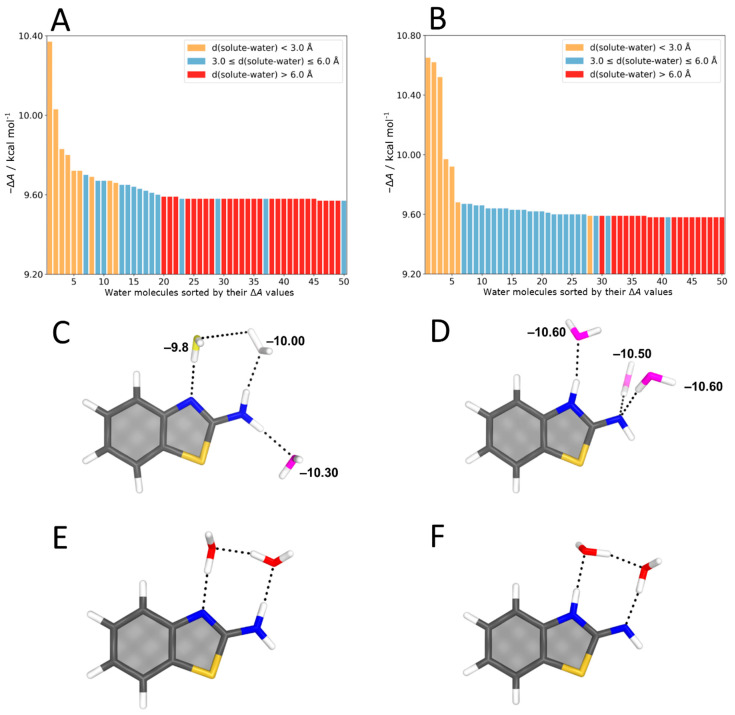
Ranking of the automatically placed water molecules around the T1 (**A**) and the T2 (**B**) conformer of 2-aminobenzothiazole. Representation of T1 (**C**) and T2 (**D**) water clusters with three explicit water molecules; water molecules are color-coded according to their free energy from pink (very favorable) to yellow (less favorable); B3LYP/def2-TZVP/D3 optimized conformers of T1-(H_2_O)_2_ (**E**) and T2-(H_2_O)_2_ (**F**).

The results of our microsolvation analysis are presented in Figure 7, where the FEBISS bar charts for both tautomers are shown. For T1, we observe six consecutive water molecules with a solute–water distance below 3 Å, of which two water molecules have a Δ*A* value lower than −10.00 kcal/mol (Figure 7A). Regarding T2, there are also six water molecules ranked in a row, with a solute–water distance smaller than 3 Å (Figure 7B), while three water molecules have a Δ*A* value lower than −10.50 kcal/mol. If we inspect these water positions in more detail (Figure 7C), we find for T1 that the most tightly bound water is a distal water with a coordination to the amine nitrogen, whereas the next two water molecules are pre-aligned to form a network between the two nitrogen nuclei to facilitate proton transfer. In the case of the trans-imino isomer T2 (Figure 7D), the first two water molecules with Δ*A* = −10.60 kcal/mol can be considered to be pre-aligned to form a water chain and to facilitate proton transfer. The third water also coordinates to the imine nitrogen, but is almost perpendicular to the aromatic ring.

Our FEBISS algorithm clearly predicts two water molecules to form an intermolecular hydrogen bonded chain to facilitate the proton transfer from the amino to the imino tautomer, and vice versa. Hence, we set up the microsolvated structures with two explicit water molecules to yield T1-(H_2_O)_2_ and T2-(H_2_O)_2_ and optimized them (Figure 7E,F). The microsolvation shifts the equilibrium by decreasing the energy difference between the two isomers Δ*E*_rel_ *= E*(T2-(H_2_O)_2_) − *E*(T1-(H_2_O)_2_) to 2.9 kcal/mol (gas phase) and 4.5 kcal/mol (implicit solvent), respectively—a finding in agreement with previously published results [49]. More strikingly, however, the predicted number of two water molecules forming a water chain and aiding the proton transfer process coincides with previous results found by trial and error [49]. Wazzan et al. reported that two water molecules minimize the barrier for proton transfer from T1 to T2, whereas chains consisting of one or three water molecules result in higher barriers.

### 4.3. Benzotriborneol–Water Complexes

Our largest test compound was (+)-*syn*-benzotriborneol, which was synthesized by Fabris et al. [48]. The structure can be characterized as a C_3_-symmetric triterpene, with a central aromatic ring and three hydroxyl groups (Scheme 1). By means of X-ray crystal structure and neutron diffraction analysis, it was proven that the molecule acts as an efficient water receptor. In total, three water molecules were found in the X-ray crystal structure of (+)-*syn*-benzotriborneol. Out of those three water molecules, one molecule is bound in the center of the molecule’s cavity, forming hydrogen bonds with the surrounding hydroxyl groups. We aimed at reproducing those experimental outcomes and subjected (+)-*syn*-benzotriborneol to our FEBISS calculation. 

**Figure 8:**
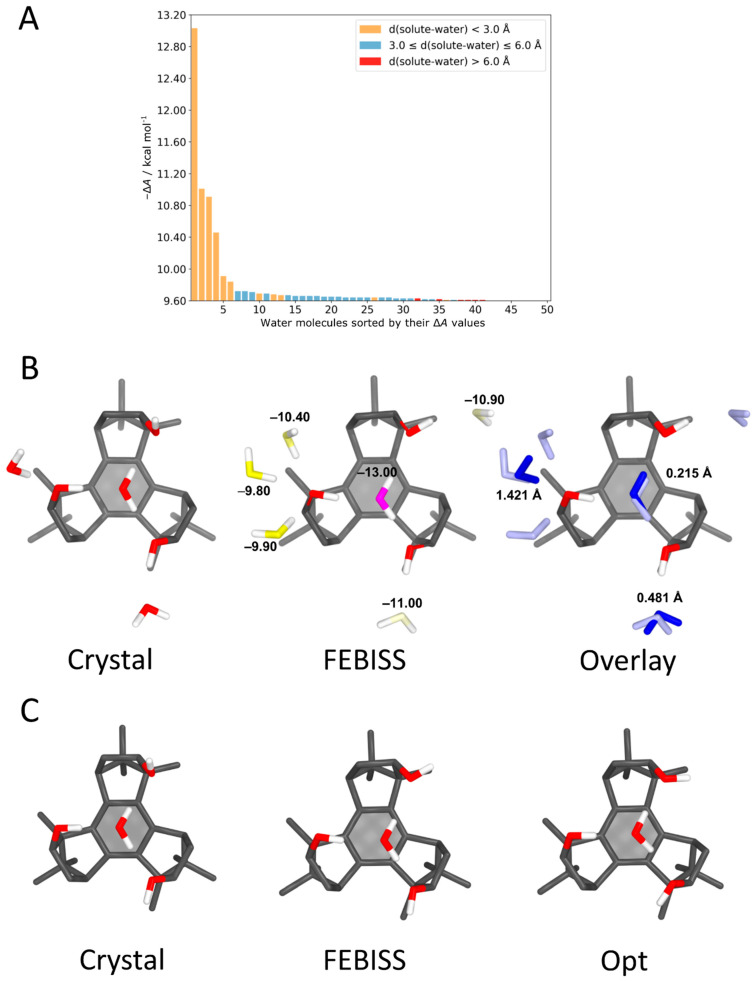
(**A**) Automated water placement of (+)-*syn*-benzotriborneol. (**B**) Comparison of the crystal structure with crystal waters (left) and our FEBISS water positions (middle) as well as an overlay of the two (right). FEBISS water positions are depicted in light blue, and crystal waters in dark blue; the depicted values indicate free energies of solvation (middle) and root mean square deviation between the crystal and the nearest FEBISS water (right). (**C**) Comparison of the crystal structure with one explicit water (left), the FEBISS water position (middle) and the B3LYP/def2-TZVP/D3 optimized (+)-*syn*-benzotriborneol-(H_2_O) cluster structure (right).

The resulting bar chart is shown in Figure 8A, where the first 50 water molecules—ranked according to their Δ*A* values—are shown. Out of those 50 water molecules, six consecutive water molecules have a solute–water distance below 3 Å. Most strikingly, we can observe a single water molecule with a Δ*A* value of around −13.00 kcal/mol, which clearly stands out from the rest of the water molecules. 

To compare the X-ray crystal structure with the FEBISS water position, we plotted both in Figure 8B. Most remarkable, our microsolvation protocol exactly predicted the position of the most strongly bound central water molecule (Δ*A* = −13.00 kcal/mol), where the water molecule and the hydroxyl groups of (+)-*syn*-benzotriborneol both act as hydrogen bond donor and acceptor. As visible from the overlay of the FEBISS and the crystal structure (Figure 8B (right)), the two water molecules are in almost perfect alignment with a root mean square deviation (RMSD) of 0.215 Å. Indeed, DFT reoptimization hardly changed the structure (Figure 8C (right)), with averaged distances of the water and the three hydroxyl groups being 1.87 Å, 1.82 Å (crystal), and 1.88 Å (FEBISS), respectively. Considering the next five predicted water positions, Figure 8B (middle) reveals that they form hydrogen bonds with the three hydroxy groups. They show also good agreement with the additional two crystal waters. The RMSD between the second most favorable FEBISS water and the crystal water is 0.481 Å, while it is 1.421 Å between the third crystal water and the nearest FEBISS water. 

### 4.4. Benzoic Acid–Water Complexes

Due to the amphiphilic nature of benzoic acid (BA), we included the molecule in our study to investigate whether our algorithm can distinguish between the hydrophilic and hydrophobic site. To assess the algorithm’s performance when working with charged molecules, we also studied the deprotonated benzoate.

**Figure 9:**
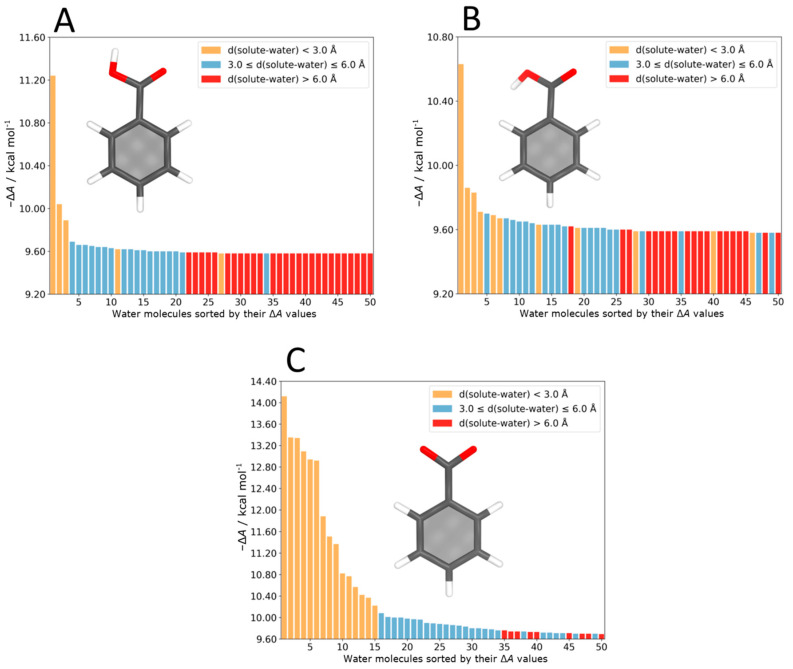
Bar chart of the FEBISS calculation for *syn*-BA (**A**) and *anti*-BA (**B**) as well as of benzoate (**C**).

The hydroxyl group of BA exists in two distinct orientations, which are denoted as *syn* and *anti,* whereas the *syn* conformation is by 6.0 kcal/mol (gas phase) and 3.2 kcal/mol (implicit solvent) lower in energy (compare Figure S4 of Supplementary Materials). This is in agreement with previous experimental and computational investigations, [53,54,55,56,58,59] where values range between 6.0 kcal/mol and 6.5 kcal/mol in the gas phase [55,56,59]. The FEBISS bar charts for *syn*-BA and *anti*-BA as well as benzoate are depicted in Figure 9A–C. All three graphs show the ranking of the first 50 automatically placed water molecules. Regarding *syn*-BA and *anti*-BA, we observe three water molecules, with a solute–water distance below 3 Å (Figure 9A). Out of those three water molecules, two show similar Δ*A* values, while one water molecule stands out with a Δ*A* value of −11.20 kcal/mol and −10.60 kcal/mol, respectively. On the contrary, the bar chart of the BA anion shows numerous water molecules with a solute–water distance lower than 3 Å (Figure 9C). In total, this includes 15 consecutive water molecules, which have Δ*A* values between −10.00 and −14.00 kcal/mol. These solvent molecules are almost exclusively located around the charged carboxylate group as visible from the initial microsolvated structures with *n* = 1, 2, 3, 6, 9, 15 displayed in Figure S5 in Supplementary Materials. Only for *n* = 15, we see water molecules being located on top of the phenyl ring.

For the two conformers of BA, we investigated the energy difference between the *syn*- and the *anti*-structure in the gas phase and in implicit solvent for various cluster sizes BA-(H_2_O)*_n_* (*n* = 0–3). When looking at the listed energy differences of the complexes in the gas phase and in implicit solvent (Table 1), distinct differences in the energies can be found—lacking a systematic pattern: for example, for *n* = 1, a relative energy difference of −9.0 kcal/mol in favor of *syn* is determined for the gas phase in agreement with previous findings of −8.9 kcal/mol, [54] but this value increases to −13.4 kcal/mol for *n* = 2 before collapsing to −2.3 kcal/mol for *n* = 3. The energy spread is less pronounced for implicit solvent calculations (2nd row, Table 1), where energy differences are consistent with the exception of *n* = 2. 

**Figure 10:**
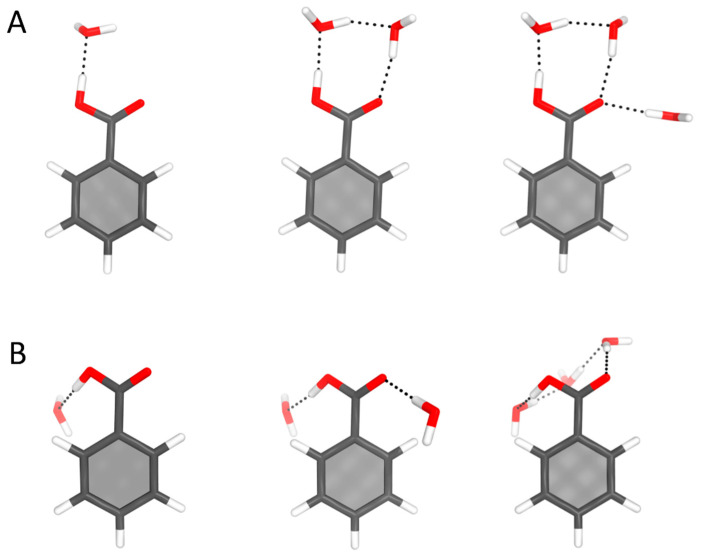
Microsolvated structures of *syn*-BA (**A**) and *anti*-BA (**B**) after B3LYP/def2-TZVP/D3 optimization in the implicit solvent.

The microsolvated clusters optimized with an additional implicit solvent model are depicted in Figure 10. The microsolvated clusters in gas phase are shown in Figure S6 in Supplementary Materials. Almost all gas phase and implicit solvent structures are remarkably similar the only exception being *syn*-BA-(H_2_O), where the water molecule forms two H-bonds to the solute in the gas phase (Figure S6) and one H-bond in the implicit solvent structure (Figure 10). However, when comparing the *syn*- and *anti*-species with *n* = 1 and *n* = 2 (Figure 10), it can be seen that the locations of the explicit solvent molecules are very different. In case of the BA-(H_2_O)_2_ even the number of hydrogen bonds differs between the *syn*- and the *anti*-conformer, which skews the energy difference. For BA-(H_2_O)_3_, however, the two structures are reasonably similar. Consequently, the *syn-anti* energy differences in the gas phase and the implicit solvent are also similar with −2.3 kcal/mol and −2.2 kcal/mol, respectively.

### 4.5. Helicene–Water Complexes

The most apolar molecule of our study is [4]-helicene, which belongs to the family of polycyclic aromatic hydrocarbons (PAHs) [90]. Such molecules and their interaction with water are of interest for studying ice-grain formation [51]. [4]-Helicene–water clusters have been previously investigated by Domingos et al., who provided experimental data on the arrangement of a single water molecule around [4]-helicene in the gas phase, using high-resolution rotational spectroscopy. The results of their study served as further benchmark in evaluating the performance of our FEBISS algorithm. 

**Figure 11:**
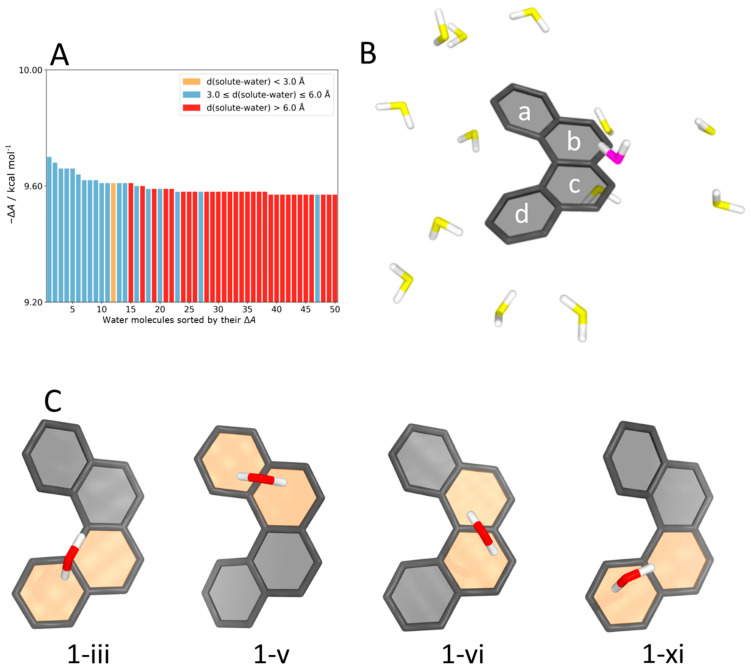
(**A**) FEBISS graph of [4]-helicene. (**B**) [4]-helicene with the 14 highest ranked water molecules as obtained from FEBISS, molecules are color-coded according to their Δ*A* from favorable (magenta) to less favorable (yellow). (**C**) Representative low energy conformation of the [4]-helicene–(H_2_O) cluster after quantum chemical optimization (B3LYP/def-TZVP/D3). The orange area highlights the aromatic ring, which interacts with the explicit water molecule. Roman numbers indicate the rank of the water position in the FEBISS bar chart.

Our automated solvent placement protocol yielded the free energy bar chart depicted in Figure 11A. We observe that none of the water molecules exceeds a Δ*A* value of −9.70 kcal/mol and that all solute–water distances correspond to values larger than 3 Å. An exception is the water ranked at position #12 with a solute–water distance of 2.9 Å, barely below 3 Å. Due to the small differences in free energy, a representation of [4]-helicene and the first 14 consecutive water molecules from our FEBISS calculation is shown in Figure 11B. It is evident that the algorithm hardly places any water molecules in close proximity to the aromatic rings, but rather locates them in the surroundings of the solute’s hydrogen atoms. 

From the FEBISS bar chart and from inspection of the positioned water molecules, we could not infer which water positions would yield low-energy monohydrated [4]-helicene clusters (denoted as [4]-helicene–(H_2_O)). Hence, we generated 14 such clusters with the water molecules ranked at position #1 to #14 in the FEBISS graph and subjected them to DFT optimizations. Several low-energy clusters were found (Table S4, Supplementary Materials) and selected low-energy conformers are depicted in Figure 11C. They are denoted as 1-iii (Δ*E* = 0.17 kcal/mol), 1-v (Δ*E* = 0.15 kcal/mol), 1-vi (Δ*E* = 0.21 kcal/mol), and 1-xi (Δ*E* = 0.00 kcal/mol), whereby the number 1 is a shorthand notation for [4]-helicene–(H_2_O) and the small Roman number specifies the rank of the water molecule in the FEBISS bar chart. All other conformers were either identical to these or enantiomers.

The relative electronic energies are very similar for all four complexes and all clusters show a similar water arrangement, with the water hydrogen atoms directed toward one of the aromatic rings. In cluster 1-iii and 1-xi, the hydrogen atoms interact with ring number c and d, respectively. In 1-v, the water is located over rings a and b, whereas in cluster 1-vi, it is located over rings b and c. These results are in agreement with reported experimental and theoretical investigations with the exception of cluster 1-iii [51].

## 5. Discussion

General Performance. We devised a computational protocol to quantum chemical microsolvation, where the location, orientation, and interaction of individual solvent molecules with the solute are automatically calculated based on MD and GIST. Our methodology provides a rational approach to generate microsolvated clusters based on physical properties. The fully automated workflow is implemented in our Python program FEBISS. The results are presented in a bar chart, where the solvent molecules are ranked according to their free energy. By simultaneously displaying the distances to the solute, the user has two criteria to choose the number of solvent molecules for a following quantum chemical calculation. The algorithm to obtain the solvent orientations is limited to water at the moment but will be extended to arbitrary solvents in the future. Consequently, as of now, our FEBISS program can only generate solute–water clusters.

Of course, the performance of this protocol intrinsically depends on the applied force field and water model used for the initial MD calculations as well as the simulation time. These factors need to be evaluated for each investigated molecule. While the generalized Amber force field (GAFF) in combination with the TIP3P water model—which we used—was shown to yield good results for small organic compounds, [91,92,93] individual molecules may profit from a more tailored force field [94]. As pointed out, the force field can easily be modified as long as the used software package generates a trajectory and topology file convertible to be readable by Cpptraj.

To obtain accurate results, it is also necessary to assign reasonable partial charges. As previously reported, RESP charges yield better results than AM1-BCC, [93] a finding we could confirm for urea (*vide infra*).

Overall, our top-down protocol to quantum chemical microsolvation yields very good agreement with available experimental and theoretical data; specific findings and limitations will be discussed for each investigated species.

Urea-Water Clusters. Urea’s strong interaction with solvent molecules in its close proximity has been recognized by spectroscopic and theoretical investigations, even though the extent of this interaction is still under debate [95]. Theoretical investigations based on MD simulations tend to find higher hydration numbers than experimental studies with up to eight hydrogen bonds [50,96,97,98]. In contrast, by dielectric electron relaxation spectroscopy Agieienko et al. found a hydration number of only 2 [92]. Our determined hydration pattern of urea suggests up to six strongly interacting water molecules, which is in agreement with previous theoretical studies [50,52,97,98]. In particular, the positions of our water molecules agree with 3D-RISM results [50] and with the areas of hydration determined by the *ab initio* MD study of Weiss and Hofer [52]. The latter study also finds a bifurcating water molecule localized between the two amide groups, which is consistent with our results. The three oxygen coordinating water molecules found in the here presented microsolvated urea complexes are in alignment with the average number of hydrogen bonds to the urea oxygen (2.07 ± 0.75) predicted by Weiss and Hofer [52]. Still, the number and position of the strongly interacting water molecules is sensitive to the parametrization scheme, in particular, to the assigned partial charges. The water placement obtained by employing AM1-BCC charges (Figure S3) predicted four water molecules interacting with the urea oxygen and one with the two amide groups. This is clearly a poorer agreement with available reference data [50,52] concerning the number and positions of the water molecules. 

Considering the dipole moment of urea in water, experimental studies found a value of *μ*_eff_ = 10.0 ± 0.1 D in infinite solution [92]. While the calculated dipole moment of urea in implicit solvent is only 6.10 D, positioning one water molecule in between the amide groups (urea–(H_2_O)) already increased *μ* to > 9 D. The addition of five more waters to yield urea–(H_2_O)_6_ had little effect on the dipole and for the large cluster, urea–(H_2_O)_13_, *μ* decreased to 2.98 D. This finding shows that a parallel alignment of solute and solvent dipole moments is beneficial [92]. It may also indicate that out of the 13 water molecules in close proximity to the solute, some might have more bulk-like properties or show high dynamics. Hence, calculating molecular properties from a single urea–(H_2_O)_13_ cluster does not yield accurate results. Explicitly describing these “bulk-like” solvent molecules by one conformation is not adequate. 

Aminobenzothiazole–Water Clusters. Concerning the amine–imine (T1 to T2) tautomerization process in ABT, our FEBISS algorithm predicted exactly two waters forming a hydrogen bonded chain and aiding the proton transfer reaction. These two solvent molecules were previously only identified by trial and error [49]. While these two bridging water molecules were correctly identified as strongly interacting solvent molecules by FEBISS, a distal amine-coordinated water was determined to have the most favorable free energy for T1 (see Figure 7). Obviously, to compare stabilities between two configurations, the microsolvated clusters T1 and T2 have to be structurally similar to obtain reliable results. Hence, the user has to select meaningful water positions based on the available data, such as solute–solvent interaction strength and location of solvent molecules.

Benzotriborneol–Water Clusters. Our FEBISS algorithm predicted the position of the most strongly bound central water molecule of the water receptor (+)-*syn*-benzotriborneol in quantitative agreement with experimental X-ray and neutron diffraction data [48]. Remarkably, not only the position of the oxygen atom but also the orientations of the hydrogens are in agreement with experiment—a finding that highlights the accuracy and reliability of our microsolvation protocol.

The positions of the next five water molecules are all in close proximity to the three hydroxyl groups as expected based on chemical intuition. Two of the solvent molecules are also in good agreement with X-ray crystal water positions (Figure 8B (right)). However, differences are expected as packing effects and symmetry mates influence the position of crystal waters and the environment in the crystal structure clearly differs from that in solution.

Benzoic Acid–Water Clusters. Concerning the microsolvation of benzoic acid, the FEBISS bar charts (Figure 9) clearly show that our algorithm is able to distinguish between charged and neutral species as indicated by the distinct water placement. The benzoate anion attracts more water molecules to aid charge compensation than the protonated BA species, which is in alignment with chemical intuition. The comparison of the microsolvated *syn*- and *anti*-conformer of BA further illustrates that in any explicit-implicit solvation model, the microsolvated structures have to be similar to be comparable in energy. In particular, the number of hydrogen bonds must be carefully examined. If it differs as, for example, in the BA-(H_2_O)_2_ cluster, it has to be evaluated whether this is inherent to the specific geometry or whether this is due to additional solvent–solvent interaction as found in our case. Still, energy differences between the *syn*- and *anti*-cluster calculated in the gas phase and in implicit solvation became very similar for three explicitly considered water molecules. As these are the only water molecules placed in close proximity to the solute (*d* < 3 Å, see Figure 9) by our FEBISS algorithm, it can be assumed that solvation effects of the COOH group are sufficiently described.

Comparing the cluster geometries, the predicted position of the most favorable water molecule in the gas phase optimized *syn*-BA-(H_2_O) complex (Figure S6)—bridging the hydroxyl and the carbonyl group—agrees with spectroscopic microwave data [57] and theoretical studies [33,96]. For the implicit solvent optimization of *syn*-BA-(H_2_O), no such bridging was found but a single hydrogen bond was formed between BA and H_2_O (Figure 10). Such a structure further suggests that a single water molecule is not sufficient to describe the microsolvation environment present in the bulk phase. Larger clusters with *n* > 2 reported in a previous study [99] showed some differences with our structures. However, these deviations can be attributed to the different methodologies to retrieve the microsolvated molecules: Krishnakumar [99] et al. used MD snapshots, whereas we use averaged highly populated positions.

Helicene–Water Clusters. Helicene was the most challenging system for our FEBISS algorithm as the interaction with water was expected to be weak due to its apolar nature. Indeed, all predicted water positions had a distance of >3 Å and their interaction with the solute was barely larger than the value for a typical bulk water (see red bars in Figure 11A). In a pragmatic approach, we generated 14 [4]-helicene–(H_2_O) clusters and obtained four low-energy conformers. When comparing our computed results with the rotational spectroscopy data from Domingos et al., [51] we can see striking similarities regarding the binding mode and position of a single water molecule toward [4]-helicene. In agreement with their data, we found the most tightly bound water molecule (cluster 1-xi) to be aligned on top of ring d and c, and also found two more conformers with the water located on top of rings a and b (1-v) and rings b and c (1-vi), respectively. Our fourth cluster, 1-iii, with the water placement similar to 1-xi was not reported by Domingos, but it remains unclear why.

Although the ranking of the water molecules according to their free energy provided little insight into which solvent molecules to choose for subsequent quantum chemical studies, the FEBISS algorithm yielded rational and reasonable starting structures for optimization. Nonetheless, we would like to point out here that the poorer performance for helicene is not a weakness of the methodology per se but can be attributed to the apolar nature of the molecule, its low solubility in water, and potentially lower accuracy of the force field for such species.

Applicability. The FEBISS protocol provides a physically sound basis to obtain microsolvated clusters. However, the assigned water locations are averaged positions. If the solute–solvent cluster is dominated by one conformation, this is a good approximation. However, depending on the investigated system and property of interest, it might be necessary to consider multiple solute conformations. In addition, it might also be necessary to subject the obtained solute–solvent cluster to additional sampling of the water positions—this time at a quantum chemical or semi-empirical level to generate a structural ensemble that may be used for Boltzmann averaging.

Still, even when only a single conformer of a microsolvated cluster is considered, DFT was shown to be well suited to describe solute–solvent interactions, [100] supporting the added value of low-cost microsolvation approaches to approximately model (bulk) solvent effects. 

## 6. Conclusions

In this paper, we presented a quantitative approach to quantum chemical microsolvation based on MD simulations and GIST analysis, which assigns water position and orientations based on physical quantities. Our protocol provides a rigorous basis as to where to locate the explicit solvent molecules, how to orient them, and how many to include, thereby allowing a rational setup of microsolvated clusters.

We demonstrated that our computational protocol is applicable to a number of different molecules ranging from biologically relevant urea and benzoic acid to apolar helicene. We retrieved solute–solvent complexes that are in very good agreement with experimental and theoretical reference data. Although currently limited to water as a solvent, we will extend our algorithm to arbitrary solvents in the future.

Our methodology can be used to set up Quantum Mechanics/Molecular Mechanics models, where the number of solvent molecules in the QM region may be determined by our FEBISS algorithm. In addition, our protocol paves the way to study explicit solute–solvent interactions in large complexes that cannot be treated by *ab initio* MD.

## Figures and Tables

**Table 1:** Relative stability differences between the microsolvated *syn*- and *anti*-conformer of BA in the gas phase (Δ*E*_gas_) and in the implicit solvent (Δ*E*_solv_). Structures were optimized with B3LYP/def2-TZVP/D3. Relative electronic energy differences are given in kcal/mol.

Structure	BA	BA-(H_2_O)	BA-(H_2_O)_2_	BA-(H_2_O)_3_
Δ*E*_gas_(*syn*-*anti*)	−6.0	−9.0	−13.4	−2.3
Δ*E*_solv_(*syn*-*anti*)	−3.2	−2.7	−6.2	−2.2

## Data Availability

Software available at https://github.com/PodewitzLab/FEBISS ((accessed on 25 February 2021)). Raw data available upon request from the authors.

## Supplementary Materials

The following are available online. Figure S1: Radial distribution functions g(r) from the 300 ns simulation trajectory of urea. Table S1: Radial distribution functions (RDFs) for certain urea-water atom pairs, calculated from our MD simulation, in comparison to previously published results. Figure S2: Urea-(H_2_O)*_n_* complexes for *n* = 1 and *n* = 6 as obtained by the FEBISS algorithm. The free energy of the solvent molecules is given in kcal/mol and they are color-coded according to this value from strongly favored (magenta) to less favored (yellow). Figure S3: FEBISS bar chart (A) and initial urea-(H_2_O)_5_ cluster when AM1-BCC charges were used in the MD simulation. Figure S4: Quantum chemically optimized structures of *syn*-BA and *anti*-BA. The relative energies were calculated with B3LYP/def2-TZVP/D3 in water modelled as implicit. Table S2: Relative energies (kcal/mol) and dipole moments (Debye) of the *syn*- and *anti*-conformer of benzoic acid calculated with B3LYP/def2-TZVP/D3 in the gas phase and in implicit solvent. Figure S5: Benzoate-(H_2_O)*_n_* clusters as obtained from the FEBISS algorithm for *n* = 1–3 water molecules (top) and *n* = 6, 9, 15 water molecules (bottom). The water molecules are color-coded according to their interaction strength from magenta (most favorable) to yellow (less favorable). Figure S6: Gas phase optimized microsolvated cluster of benzoic acid in the *syn*- and *anti*-conformation. Table S3. Relative energies (kcal/mol) and dipole moments (Debye) of tautomer T1 and T2 calculated with B3LYP-D3(BJ)/def2-TZVP for in the gas phase and in implicit solvent. Figure S7a: Structural overview of the B3LYP/def2-TZVP/D3 optimized microsolvated clusters of T1-(H_2_O)*_n_* and T2(H_2_O)*_n_* with *n* = 1–3 water molecules. The initial water placement was done with our FEBISS algorithm. Figure S7b: Structural overview of the B3LYP/def2-TZVP/D3 optimized microsolvated clusters of T1-(H_2_O)*_n_* and T2-(H_2_O)*_n_* with *n* = 4–6 water molecules. The initial water placement was done with our FEBISS algorithm. Table S4. Relative energies in kcal/mol for the first 14 [4]-helicene-(H_2_O) clusters.

## Sample Availability

Samples of the compunds are not available from the authors.

## References

[1] Sherwood J., Clark J.H., Fairlamb I.J.S., Slattery J.M. (2019). Solvent effects in palladium catalysed cross-coupling reactions. Green Chem..

[2] Pirrung M.C. (2006). Acceleration of Organic Reactions through Aqueous Solvent Effects. Chem. Eur. J..

[3] Reichardt C. (2007). Solvents and Solvent Effects: An Introduction. Org. Process. Res. Dev..

[4] Reichardt C., Welton T. (2010). Solute-Solvent Interactions. Solvents and Solvent Effects in Organic Chemistry.

[5] Elmi A., Cockroft S.L. (2021). Quantifying Interactions and Solvent Effects Using Molecular Balances and Model Complexes. Acc. Chem. Res..

[6] Varghese J.J., Mushrif S.H. (2019). Origins of complex solvent effects on chemical reactivity and computational tools to investigate them: A review. React. Chem. Eng..

[7] Sunoj R.B., Anand M. (2012). Microsolvated transition state models for improved insight into chemical properties and reaction mechanisms. Phys. Chem. Chem. Phys..

[8] Pliego J.R., Riveros J.M. (2020). Hybrid discrete-continuum solvation methods. Wiley Interdiscip. Rev. Comput. Mol. Sci..

[9] Cramer C.J., Truhlar D.G. (2008). A Universal Approach to Solvation Modeling. Acc. Chem. Res..

[10] Tomasi J., Mennucci B., Cammi R. (2005). Quantum Mechanical Continuum Solvation Models. Chem. Rev..

[11] Marenich A.V., Cramer C.J., Truhlar D.G. (2009). Universal Solvation Model Based on Solute Electron Density and on a Continuum Model of the Solvent Defined by the Bulk Dielectric Constant and Atomic Surface Tensions. J. Phys. Chem. B.

[12] Klamt A. (2011). The COSMO and COSMO-RS solvation models. Wiley Interdiscip. Rev. Comput. Mol. Sci..

[13] Marx D., Hutter J. (2009). Ab Initio Molecular Dynamics: Basic Theory and Advanced Methods.

[14] Hutter J. (2011). Car-Parrinello molecular dynamics. Wiley Interdiscip. Rev. Comput. Mol. Sci..

[15] Kühne T.D. (2014). Second generation Car-Parrinello molecular dynamics. Wiley Interdiscip. Rev. Comput. Mol. Sci..

[16] Kelly C.P., Cramer C.J., Truhlar D.G. (2006). Adding Explicit Solvent Molecules to Continuum Solvent Calculations for the Calculation of Aqueous Acid Dissociation Constants. J. Phys. Chem. A.

[17] Thar J., Zahn S., Kirchner B. (2008). When Is a Molecule Properly Solvated by a Continuum Model or in a Cluster Ansatz? A First-Principles Simulation of Alanine Hydration. J. Phys. Chem. B.

[18] Pliego J.A.J.R., Riveros J.M. (2001). The Cluster−Continuum Model for the Calculation of the Solvation Free Energy of Ionic Species. J. Phys. Chem. A.

[19] Bryantsev V.S., Diallo M.S., Iii W.A.G. (2008). Calculation of Solvation Free Energies of Charged Solutes Using Mixed Cluster/Continuum Models. J. Phys. Chem. B.

[20] Marenich A.V., Ding W., Cramer C.J., Truhlar N.G. (2012). Resolution of a Challenge for Solvation Modeling: Calculation of Dicarboxylic Acid Dissociation Constants Using Mixed Discrete–Continuum Solvation Models. J. Phys. Chem. Lett..

[21] Zins E.-L. (2020). Microhydration of a Carbonyl Group: How does the Molecular Electrostatic Potential (MESP) Impact the Formation of (H_2_O)*_n_*:(R_2_C═O)Complexes?. J. Phys. Chem. A.

[22] Gadre S.R., Babu K., Rendell A.P. (2000). Electrostatics for Exploring Hydration Patterns of Molecules. 3. Uracil. J. Phys. Chem. A.

[23] Kalai C., Alikhani M.E., Zins E.L. (2018). The molecular electrostatic potential analysis of solutes and water clusters: A straightforward tool to predict the geometry of the most stable micro-hydrated complexes of beta-propiolactone and formamide. Theor. Chem. Acc..

[24] Sure R., El Mahdali M., Plajer A., Deglmann P. (2021). Towards a converged strategy for including microsolvation in reaction mechanism calculations. J. Comput. Mol. Des..

[25] Lanza G., Chiacchio M.A. (2020). The water molecule arrangement over the side chain of some aliphatic amino acids: A quantum chemical and bottom-up investigation. Int. J. Quantum Chem..

[26] Simm G.N., Türtscher P.L., Reiher M. (2020). Systematic microsolvation approach with a cluster-continuum scheme and conformational sampling. J. Comput. Chem..

[27] Basdogan Y., Groenenboom M.C., Henderson E., De S., Rempe S.B., Keith J.A. (2019). Machine Learning-Guided Approach for Studying Solvation Environments. J. Chem. Theory Comput..

[28] Jesus W.S., Prudente F.V., Marques J.M.C., Pereira F.B. (2021). Modeling microsolvation clusters with electronic-structure calculations guided by analytical potentials and predictive machine learning techniques. Phys. Chem. Chem. Phys..

[29] Kongsted J., Mennucci B., Coutinho K., Canuto S. (2010). Solvent effects on the electronic absorption spectrum of camphor using continuum, discrete or explicit approaches. Chem. Phys. Lett..

[30] Mensch C., Bultinck P., Johannessen C. (2019). The effect of protein backbone hydration on the amide vibrations in Raman and Raman optical activity spectra. Phys. Chem. Chem. Phys..

[31] Da Silva E.F., Svendsen H.F., Merz K.M. (2009). Explicitly Representing the Solvation Shell in Continuum Solvent Calculations. J. Phys. Chem. A.

[32] Nocito D., Beran G.J.O. (2017). Averaged Condensed Phase Model for Simulating Molecules in Complex Environments. J. Chem. Theory Comput..

[33] Karimova N.V., Luo M., Grassian V.H., Gerber R.B. (2020). Absorption spectra of benzoic acid in water at different pH and in the presence of salts: Insights from the integration of experimental data and theoretical cluster models. Phys. Chem. Chem. Phys..

[34] Basdogan Y., Maldonado A.M., Keith J.A. (2020). Advances and challenges in modeling solvated reaction mechanisms for renewable fuels and chemicals. Wiley Interdiscip. Rev. Comput. Mol. Sci..

[35] Bodnarchuk M.S., Viner R., Michel J., Essex J.W. (2014). Strategies to Calculate Water Binding Free Energies in Protein–Ligand Complexes. J. Chem. Inf. Model..

[36] Spyrakis F., Ahmed M.H., Bayden A.S., Cozzini P., Mozzarelli A., Kellogg G.E. (2017). The Roles of Water in the Protein Matrix: A Largely Untapped Resource for Drug Discovery. J. Med. Chem..

[37] Bucher D., Stouten P.F., Triballeau N. (2018). Shedding Light on Important Waters for Drug Design: Simulations versus Grid-Based Methods. J. Chem. Inf. Model..

[38] Rudling A., Orro A., Carlsson J. (2018). Prediction of Ordered Water Molecules in Protein Binding Sites from Molecular Dynamics Simulations: The Impact of Ligand Binding on Hydration Networks. J. Chem. Inf. Model..

[39] Jukic M., Konc J., Janezic D., Bren U. (2020). ProBiS H_2_O MD Approach for Identification of Conserved Water Sites in Protein Structures for Drug Design. ACS Med. Chem. Lett..

[40] Kearney B.M., Schwabe M., Marcus K.C., Roberts D.M., DeChene M., Swartz P., Mattos C. (2020). DRoP: Automated detection of conserved solvent-binding sites on proteins. Proteins Struct. Funct. Bioinform..

[41] Ramsey S., Nguyen C., Salomon-Ferrer R., Walker R.C., Gilson M.K., Kurtzman T. (2016). Solvation thermodynamic mapping of molecular surfaces in AmberTools: GIST. J. Comput. Chem..

[42] Nguyen C.N., Young T.K., Gilson M.K. (2012). Grid inhomogeneous solvation theory: Hydration structure and thermodynamics of the miniature receptor cucurbit[7]uril. J. Chem. Phys..

[43] Nguyen C.N., Cruz A., Gilson M.K., Kurtzman T. (2014). Thermodynamics of Water in an Enzyme Active Site: Grid-Based Hydration Analysis of Coagulation Factor Xa. J. Chem. Theory Comput..

[44] Loeffler J.R., Schauperl M., Liedl K.R. (2019). Hydration of Aromatic Heterocycles as an Adversary of pi-Stacking. J. Chem. Inf. Model..

[45] Loeffler J.R., Fernández-Quintero M.L., Schauperl M., Liedl K.R. (2020). STACKED – Solvation Theory of Aromatic Complexes as Key for Estimating Drug Binding. J. Chem. Inf. Model..

[46] Schauperl M., Czodrowski P., Fuchs J.E., Huber R.G., Waldner B.J., Podewitz M., Kramer C., Liedl K.R. (2017). Binding Pose Flip Explained via Enthalpic and Entropic Contributions. J. Chem. Inf. Model..

[47] Schauperl M., Podewitz M., Ortner T.S., Waibl F., Thoeny A., Loerting T., Liedl K.R. (2017). Balance between hydration enthalpy and entropy is important for ice binding surfaces in Antifreeze Proteins. Sci. Rep..

[48] Fabris F., De Lucchi O., Nardini I., Crisma M., Mazzanti A., Mason S.A., Lemée-Cailleau M.-H., Scaramuzzo F.A., Zonta C. (2012). (+)-*syn*-Benzotriborneol an enantiopure C_3_-symmetric receptor for water. Org. Biomol. Chem..

[49] Wazzan N., Safi Z., Al-Barakati R., Al-Qurashi O., Al-Khateeb L. (2019). DFT investigation on the intramolecular and intermolecular proton transfer processes in 2-aminobenzothiazole (ABT) in the gas phase and in different solvents. Struct. Chem..

[50] Ishida T., Rossky P.J., Castner E.W. (2004). A Theoretical Investigation of the Shape and Hydration Properties of Aqueous Urea: Evidence for Nonplanar Urea Geometry. J. Phys. Chem. B.

[51] Domingos S.R., Martin K., Avarvari N., Schnell M. (2019). Water Docking Bias in 4 Helicene. Angew. Chem. Int. Ed..

[52] Weiss A.K.H., Hofer T.S. (2013). Urea in aqueous solution studied by quantum mechanical charge field-molecular dynamics (QMCF-MD). Mol. BioSyst..

[53] Bruno G., Randaccio L. (1980). A refinement of the benzoic acid structure at room temperature. Acta Crystallogr. Sect. B Struct. Crystallogr. Cryst. Chem..

[54] Nagy P.I., Smith D.A., Alagona G., Ghio C. (1994). *Ab initio* studies of free and monohydrated carboxylic acids in the gas phase. J. Phys. Chem..

[55] Stepanian S., Reva I., Radchenko E., Sheina G. (1996). Infrared spectra of benzoic acid monomers and dimers in argon matrix. Vib. Spectrosc..

[56] Godfrey P.D., McNaughton D. (2013). Structural studies of aromatic carboxylic acids via computational chemistry and microwave spectroscopy. J. Chem. Phys..

[57] Schnitzler E.G., Jäger W. (2014). The benzoic acid–water complex: A potential atmospheric nucleation precursor studied using microwave spectroscopy and *ab initio* calculations. Phys. Chem. Chem. Phys..

[58] Onda M., Asai M., Takise K., Kuwae K., Hayami K., Kuroe A., Mori M., Miyazaki H., Suzuki N., Yamaguchi I. (1999). Microwave spectrum of benzoic acid. J. Mol. Struct..

[59] Aarset K., Page E.M., Rice D.A. (2006). Molecular Structures of Benzoic Acid and 2-Hydroxybenzoic Acid, Obtained by Gas-Phase Electron Diffraction and Theoretical Calculations. J. Phys. Chem. A.

[60] Lazaridis T. (1998). Inhomogeneous Fluid Approach to Solvation Thermodynamics. 1. Theory. J. Phys. Chem. B.

[61] Roe D.R., Cheatham T.E. (2013). PTRAJ and CPPTRAJ: Software for Processing and Analysis of Molecular Dynamics Trajectory Data. J. Chem. Theory Comput..

[62] Jorgensen W.L., Chandrasekhar J., Madura J.D., Impey R.W., Klein M.L. (1983). Comparison of simple potential functions for simulating liquid water. J. Chem. Phys..

[63] Salomon-Ferrer R., Case D.A., Walker R.C. (2013). An overview of the Amber biomolecular simulation package. Wiley Interdiscip. Rev. Comput. Mol. Sci..

[64] Case D.A., Ben-Shalom I.Y., Brozell S.R., Cerutti D.S., Cheatham I.T.E., Cruzeiro V.W.D., Darden T.A., Duke R.E., Ghoreishi D., Gilson M.K. (2018). AMBER 2018.

[65] The PyMOL Molecular Graphics System, Version 1.8. https://sourceforge.net/projects/pymol/files/pymol/1.8/.

[66] https://github.com/PodewitzLab/FEBISS.git.

[67] https://github.com/liedllab/gigist.

[68] Kraml J., Kamenik A.S., Waibl F., Schauperl M., Liedl K.R. (2019). Solvation Free Energy as a Measure of Hydrophobicity: Application to Serine Protease Binding Interfaces. J. Chem. Theory Comput..

[69] Frisch M.J., Trucks G.W., Schlegel H.B., Scuseria G.E., Robb M.A., Cheeseman J.R., Scalmani G., Barone V., Petersson G.A., Nakatsuji H. (2016). Gaussian 16 Rev. C.01.

[70] Bayly C.I., Cieplak P., Cornell W., Kollman P.A. (1993). A well-behaved electrostatic potential based method using charge restraints for deriving atomic charges: The RESP model. J. Phys. Chem..

[71] Jakalian A., Jack D.B., Bayly C.I. (2002). Fast, efficient generation of high-quality atomic charges. AM1-BCC model: II. Parameterization and validation. J. Comput. Chem..

[72] Wang J., Wolf R.M., Caldwell J.W., Kollman P.A., Case D.A. (2004). Development and testing of a general amber force field. J. Comput. Chem..

[73] Wallnoefer H.G., Liedl K.R., Fox T. (2011). A challenging system: Free energy prediction for factor Xa. J. Comput. Chem..

[74] Salomon-Ferrer R., Götz A.W., Poole D., Le Grand S., Walker R.C. (2013). Routine Microsecond Molecular Dynamics Simulations with AMBER on GPUs. 2. Explicit Solvent Particle Mesh Ewald. J. Chem. Theory Comput..

[75] Adelman S.A., Doll J.D. (1976). Generalized Langevin Equation Approach for Atom-Solid-Surface Scattering: General Formulation for Classical Scattering off Harmonic Solids. J. Chem. Phys..

[76] Darden T., York D., Pedersen L. (1993). Particle mesh Ewald: An *N*·log(*N*) method for Ewald sums in large systems. J. Chem. Phys..

[77] Ryckaert J.-P., Ciccotti G., Berendsen H.J.C. (1977). Numerical integration of the cartesian equations of motion of a system with constraints: Molecular dynamics of n-alkanes. J. Comput. Phys..

[78] Kovalenko A., Hirata F. (1999). Self-consistent description of a metal–water interface by the Kohn–Sham density functional theory and the three-dimensional reference interaction site model. J. Chem. Phys..

[79] Group C.C. (2018). Molecular Operating Environment.

[80] Furche F., Ahlrichs R., Hättig C., Klopper W., Sierka M., Weigend F. (2014). Turbomole. Wiley Interdiscip. Rev. Comput. Mol..

[81] TURBOMOLE V7.3 2018, a Development of University of Karlsruhe and Forschungszentrum Karlsruhe GmbH, 1989–2007, TURBOMOLE GmbH, Since 2007. http://www.turbomole.com/.

[82] Becke A.D. (1993). Density-Functional Thermochemistry III. The Role of Exact Exchange. J. Chem. Phys..

[83] Lee C., Yang W., Parr R.G. (1988). Development of the Colle-Salvetti correlation-energy formula into a functional of the electron density. Phys. Rev. B.

[84] Becke A.D. (1988). Density-functional exchange-energy approximation with correct asymptotic behavior. Phys. Rev. A.

[85] Weigend F., Ahlrichs R. (2005). Balanced Basis Sets of Split Valence, Triple Zeta Valence and Quadruple Zeta Valence Quality For H to Rn: Design and Assessment of Accuracy. Phys. Chem. Chem. Phys..

[86] Grimme S., Antony J., Ehrlich S., Krieg H. (2010). A consistent and accurate *ab initio* parametrization of density functional dispersion correction (DFT-D) for the 94 elements H-Pu. J. Chem. Phys..

[87] Grimme S., Ehrlich S., Goerigk L. (2011). Effect of the damping function in dispersion corrected density functional theory. J. Comput. Chem..

[88] Klamt A., Schüürmann G. (1993). COSMO: A new approach to dielectric screening in solvents with explicit expressions for the screening energy and its gradient. J. Chem. Soc. Perkin Trans. 2.

[89] Schäfer A., Klamt A., Sattel D., Lohrenz J.C.W., Eckert F. (2000). COSMO Implementation in TURBOMOLE: Extension of an efficient quantum chemical code towards liquid systems. Phys. Chem. Chem. Phys..

[90] Hirschfeld F.L., Schmidt G.M.J., Sandler S. (1963). 398.The Structure of Overcrowded Aromatic Compounds. Part VI. The Crystal Structure of Benzo[C]phenanthrene and of 1,12-Dimethylbenzo[C]phenanthrene. J. Chem. Soc..

[91] Mobley D.L., Dumont É., Chodera J.D., Dill K.A. (2007). Comparison of Charge Models for Fixed-Charge Force Fields: Small-Molecule Hydration Free Energies in Explicit Solvent. J. Phys. Chem. B.

[92] Mobley D.L., Bayly C.I., Cooper M.D., Shirts M.R., Dill K.A. (2009). Small Molecule Hydration Free Energies in Explicit Solvent: An Extensive Test of Fixed-Charge Atomistic Simulations. J. Chem. Theory Comput..

[93] Shivakumar D., Deng Y., Roux B. (2009). Computations of Absolute Solvation Free Energies of Small Molecules Using Explicit and Implicit Solvent Model. J. Chem. Theory Comput..

[94] Bunken C., Reiher M. (2020). Self-Parametrizing System-Focused Atomistic Models. J. Chem. Theory Comput..

[95] Agieienko V., Buchner R. (2015). Urea hydration from dielectric relaxation spectroscopy: Old findings confirmed, new insights gained. Phys. Chem. Chem. Phys..

[96] Åstrand P.-O., Wallqvist A., Karlström G., Linse P. (1991). Properties of Urea–Water Solvation Calculated from a New *ab initio* Polarizable Intermolecular Potential. J. Chem. Phys..

[97] Kallies B. (2001). Coupling of solvent and solute dynamics—Molecular dynamics simulations of aqueous urea solutions with different intramolecular potentials. Phys. Chem. Chem. Phys..

[98] Stumpe M.C., Grubmüller H. (2007). Aqueous Urea Solutions: Structure, Energetics, and Urea Aggregation. J. Phys. Chem. B.

[99] Krishnakumar P., Maity D.K. (2017). Microhydration of a benzoic acid molecule and its dissociation. New J. Chem..

[100] Chen J., Chan B., Shao Y., Ho J. (2020). How accurate are approximate quantum chemical methods at modelling solute–solvent interactions in solvated clusters?. Phys. Chem. Chem. Phys..

