# Supplementary material for: Quantum Chemical Microsolvation by Automated Water Placement
Source: Molecules. 2021 Mar 23;26(6):1793. doi: 10.3390/molecules26061793 (PMC8005176; doi:10.3390/molecules26061793)
Supplement: Supplementary file 1 [file molecules-26-01793-s001.pdf]

Supplementary Material

To

"Quantum Chemical Microsolvation by Automated Solvent Placement"

by

Miguel Steiner,[1,2] Tanja Holzknecht,[1] Michael Schauperl,[1] Maren Podewitz*[1]

[1]Institute of General, Inorganic and Theoretical Chemistry, University of Innsbruck, Innrain 80-82, 6020 Innsbruck, Austria

[2]Present address: Laboratorium für Physikalische Chemie, ETH Zürich, Vladimir-Prelog-Weg 2, 8093, Zürich, Switzerland

E-Mail: maren.podewitz@uibk.ac.at

Urea

Analysis of Radial Distribution Functions

We computed radial distribution functions (RDFs) from our simulation trajectory (Figure S1). This was achieved by calculating the probability of finding specific urea-water atom pairs in a certain distance (*r*). In Table S1, the distances *r* for the maximum RDF values are presented and compared to previously published *ab initio* Molecular Dynamics (MD)[1] and 3D Reference Interaction Site Model – Self Consistent Field (3D-RISM-SCF)[2] results. For our MD simulation, we observe a maximum RDF at 1.88 Å for the O_{urea}-H_{water} distribution and a distance maximum of 3.63 Å for the C_{urea}-O_{water} distribution. These results agree with the findings of Weiss *et al.*[1] and Ishida *et al.*,[2] who observed maxima at 2.02 Å and 1.70 Å for the O_{urea}-H_{water} distribution and 3.95 Å and 3.90 Å for the C_{urea}-O_{water} distribution. Also, the O_{urea}-O_{water} distance of 2.73 Å correlates well with the RDFs retrieved from the *ab initio* calculations. The largest differences between our MD simulation and the *ab initio* MD simulations is the maximum of the N_{urea}-O_{water} distribution. While our trajectory shows a maximum at 2.88 Å, the *ab initio* MD simulations predict distances in the range from 3.38 to 3.90 Å. Considering the differences in the underlying principles the force field based MD simulations and the *ab initio* calculations, we were not expecting identical results for both methodologies. However, we observed a good agreement for the solute-solvent distances to conclude that our classical MD simulation provides a reliable input for the FEBISS water placement algorithm.

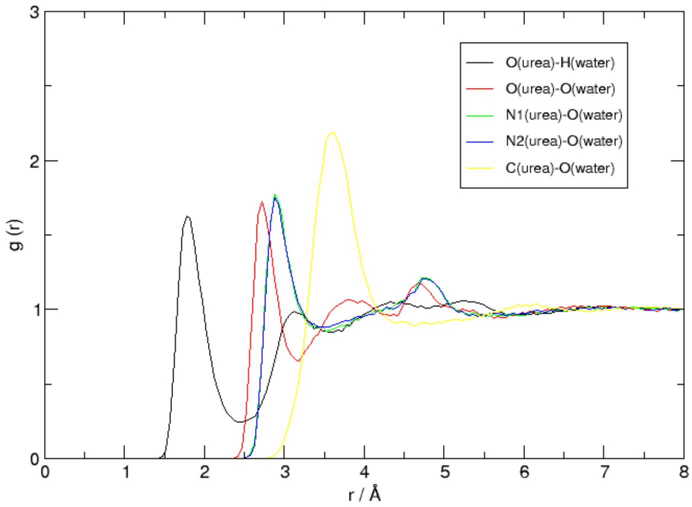

Figure S1. Radial distribution functions g(r) from the 300 ns simulation trajectory of urea.

Table S1. Radial distribution functions (RDFs) for certain urea-water atom pairs, calculated from our MD simulation, in comparison to previously published results.

	**Maxima of RDFs**		
r (Å)	This study	Weiss et al.[1]	Ishida et al.[2]
O_{urea}-H_{water}	1.88	2.02	1.70
O_{urea}-O_{water}	2.73	2.94	2.97
N_{urea}-O_{water}	2.88	3.38	~3.90
C_{urea}-O_{water}	3.63	3.95	~3.90

Water Placement for Urea

Figure S2 depicts the water placement obtained with our FEBISS algorithm when RESP charges were used for the parametrization.

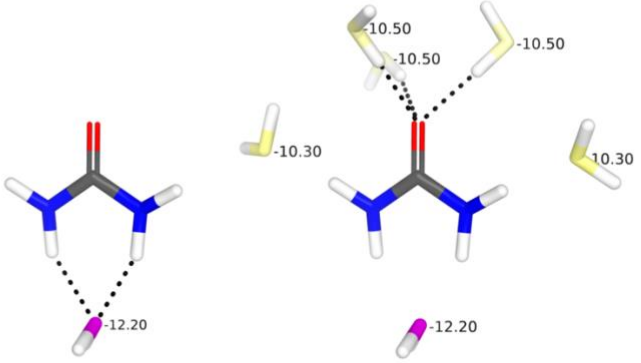

Figure S2. Urea-$(H_2O)_n$ complexes for n=1 and n=6 as obtained by the FEBISS algorithm. The free energy of the solvent molecules is given in kcal/mol and they are color-coded according to this value from strongly favored (magenta) to less favored (yellow).

For sake of comparison we also used AM1-BCC[3] charges for the urea parametrization. As visible from Figure S3, the FEBISS bar chart and the water placement show distinct differences to the parametrization with RESP charges.[4] Clearly, five water molecules display similar free energies < -10.35 kcal/mol. Out of these waters, four interact with the urea oxygen. This finding is in poorer agreement with *ab initio* reference calculations.[1]

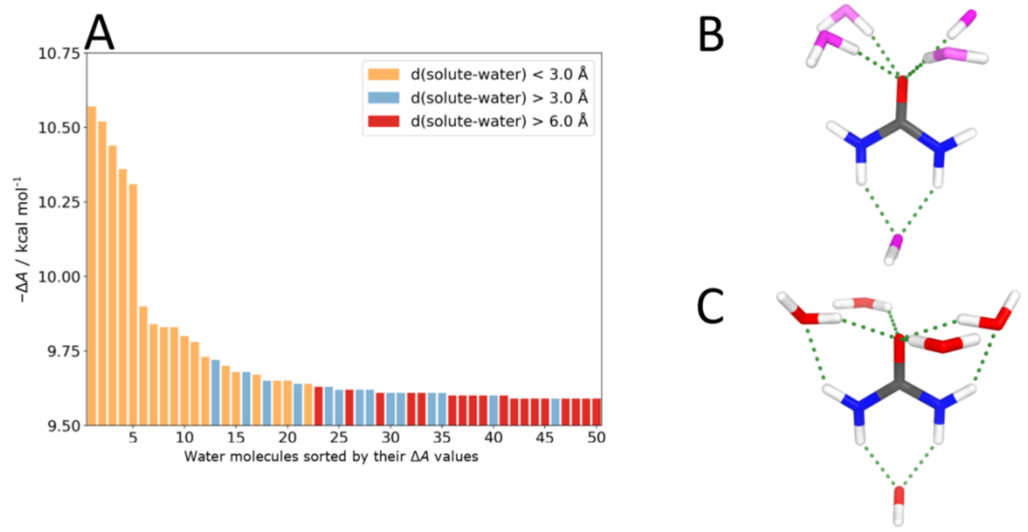

Figure S3. FEBISS bar chart (A) and initial urea-$(H_2O)_5$ cluster when AM1-BCC charges were used in the MD simulation.

Benzoic acid

Relative Stability of Benzoic Acid Conformers

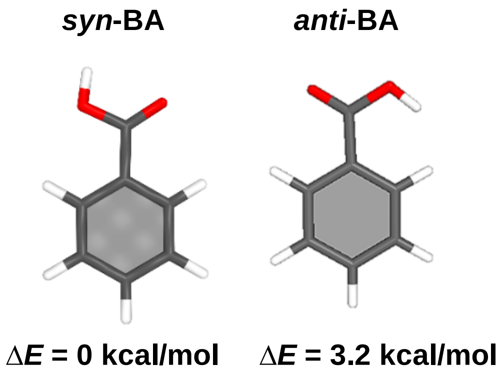

Figure S4. Quantum chemically optimized structures of *syn*-BA and *anti*-BA. The relative energies were calculated with B3LYP/def2-TZVP/D3 in water modelled as implicit.

Table S2. Relative energies (kcal/mol) and dipole moments (Debye) of the *syn* and *anti*-conformer of benzoic acid calculated with B3LYP/def2-TZVP/D3 in the gas phase and in implicit solvent.

		***syn*-BA**			***anti*-BA**		
	**ε**	**μ**	**Δ*E***	**Δ*G***	**μ**	**Δ*E***	**Δ*G***
Gas	-	2.07	0	0	4.86	6.0	5.8
Water	78.5	3.01	0	0	6.78	3.2	1.8

Microsolvated Benzoate and BA Clusters

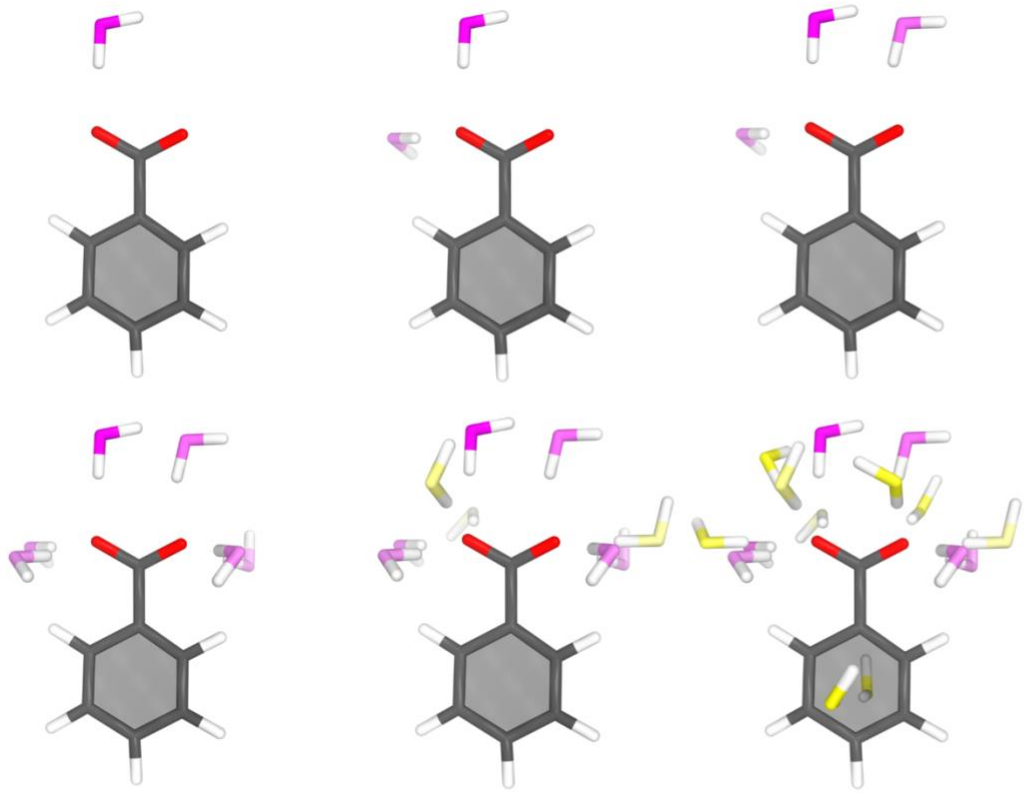

Figure S5. Benzoate-$(H_2O)_n$ clusters as obtained from the FEBISS algorithm for n=1-3 water molecules (top) and n=6, 9, 15 water molecules (bottom). The water molecules are color-coded according to their interaction strength from magenta (most favourable) to yellow (less favourable).

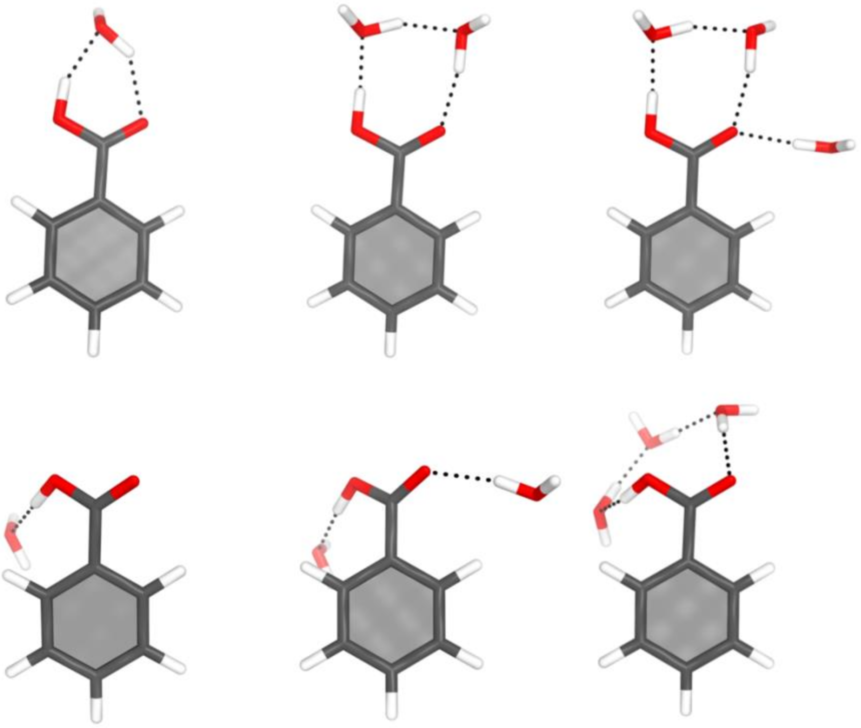

Figure S6. Gas phase optimized microsolvated cluster of benzoic acid in the *syn*- and *anti*-conformation.

Aminobenzothiazole

Table S3. Relative energies (kcal/mol) and dipole moments (Debye) of tautomer T1 and T2 calculated with B3LYP-D3(BJ)/def2-TZVP for in the gas phase and in implicit solvent.

		**T1**			**T2**		
	**ε**	**μ**	**Δ*E***	**Δ*G***	**μ**	**Δ*E***	**Δ*G***
Gas	-	2.07	0	0	2.05	5.4	5.3
Water	78.5	3.29	0	0	3.20	5.6	5.8

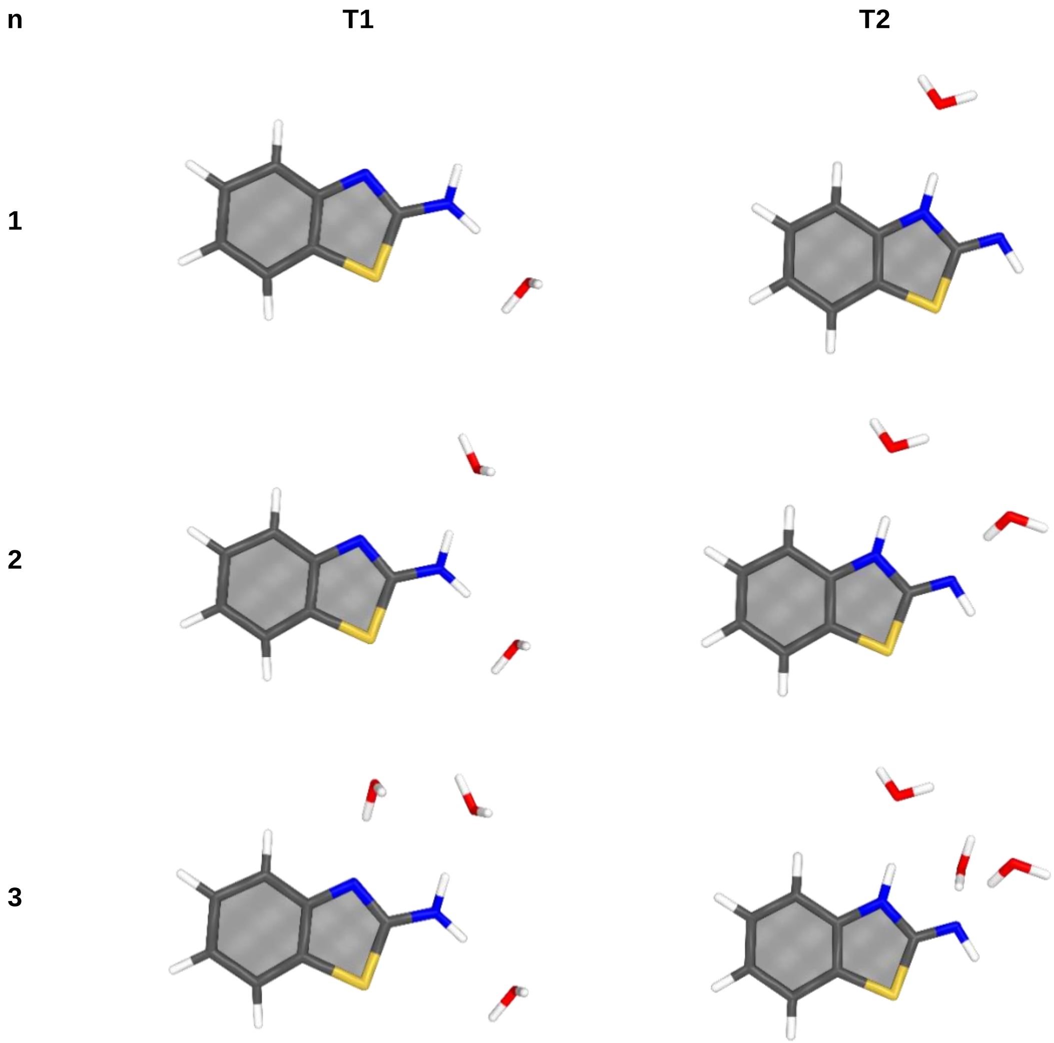

Figure S7a. Structural overview of the B3LYP/def2-TZVP/D3 optimized microsolvated clusters of T1-$(H_2O)_n$ and T2$(H_2O)_n$ with n=1-3 water molecules. The initial water placement was done with our FEBISS algorithm.

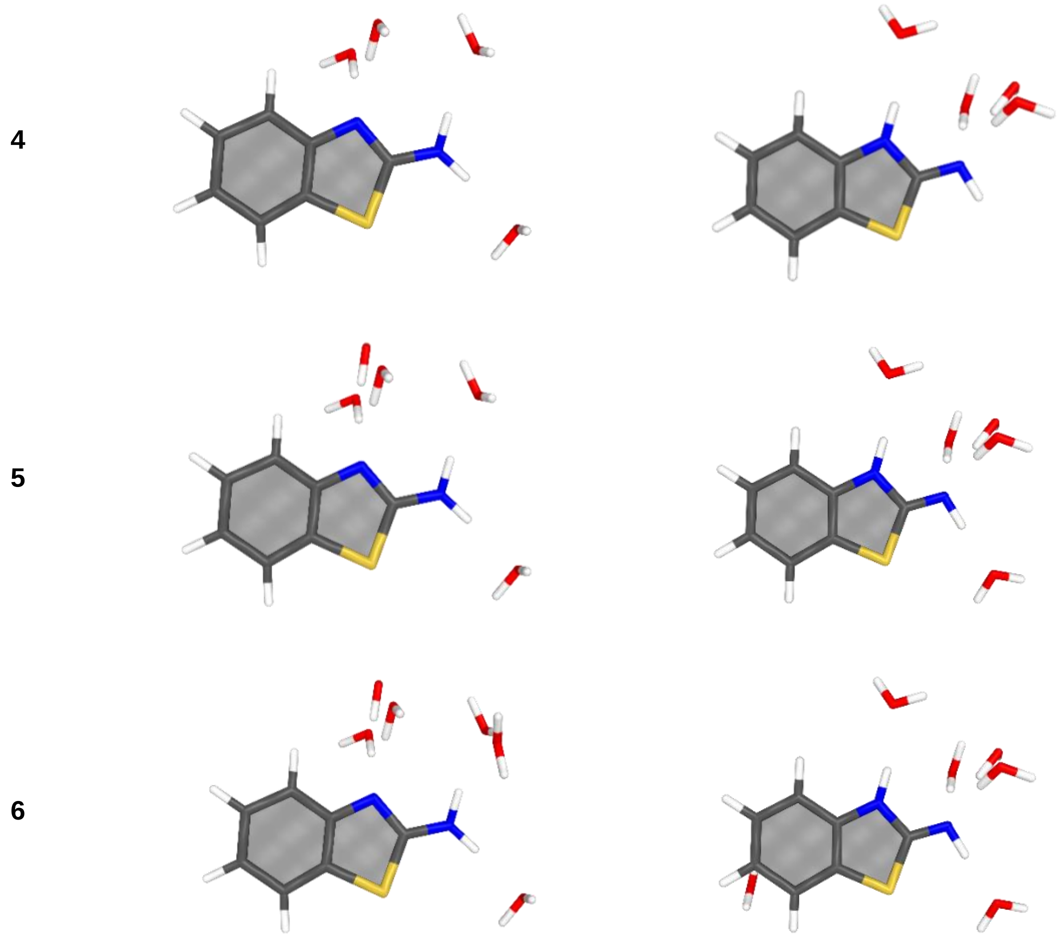

Figure S7b. Structural overview of the B3LYP/def2-TZVP/D3 optimized microsolvated clusters of T1-$(H_2O)_n$ and T2$(H_2O)_n$ with n=4-6 water molecules. The initial water placement was done with our FEBISS algorithm.

Helicene

Relative energies for the 14 [4]-helicene-(H_2O) clusters retrieved with the FEBISS algorithm and optimized with B3LYP and BP86,[5-6] respectively, are displayed in Table S4. The resulting clusters are termed 1-i to 1-xiv, where the Roman number indicates the rank of the water molecule in the FEBISS graph and "1" is a short hand notation for [4]-helicene-(H_2O).

Table S4. Relative energies in kcal/mol for the first 14 [4]-helicene-(H_2O) clusters.

Position[a]	B3LYP/def2-TZVP/D3	BP86/def2-TZVP/D3
i	0.21	0.22
ii	3.38	3.79
iii	0.17	0.20
iv	3.60	0.16
v	0.15	0.22
vi	0.21	0.23
vii	0.16	0.17
viii	0.00	0.01
ix	2.69	3.26
x	0.15	0.22
xi	0.00	0.00
xii	0.17	0.24
xiii	3.63	4.07
xiv	3.30	3.71

[a] Position refers to the rank of the water molecule in the FEBISS chart.

Considering the resulting clusters, some structures are identical such as 1-xi and 1-viii or very similar such structure 1-xii and 1-v, while others are enantiomers such as 1-v and 1-x or 1-vi and 1-i or 1-vii and 1-iii. The energy difference of 0.01 kcal/mol between 1-iii and 1-vii arises due to numerical approximation.

It can also be seen in Table S4 that both density functionals, B3LYP and BP86, yield very similar results for the low energy conformers. A notable exception is cluster 1-iv, where the B3LYP calculation resulted in a much higher relative energy due to a distal water location. In the BP86 calculation, however, the water is located over ring b and c (similar to 1-v).

FEBISS Shorthand Manual

For a more detailed FEBISS manual the reader is referred to the Github repository, where FEBISS can be downloaded free of charge (https://github.com/PodewitzLab/FEBISS.git).

Installation

The FEBISS python package is expected to run on Linux systems and requires Python3 and only basic python packages, which will be automatically installed when installing FEBISS via pip. It can be installed either directly via PyPI with

```
$ pip install febiss
```

or after downloading the source code from our Github repository with

```
$ git clone <febiss-repo>
$ pip install ./febiss
```

C++ Dependencies

To run analyses of trajectories the open-source software CPPTRAJ modified with the GIGIST repository is used. These dependencies are not necessary for the installation of FEBISS, but rather FEBISS provides a script to set-up these dependencies via

```
$ febiss_setup
```

Please be aware that CPPTRAJ may require libraries that cannot be installed via FEBISS but must be installed by the user first. CPPTRAJ makes use of the following libraries:

- NetCDF
- BLAS
- LAPACK
- Gzip
- Bzip2
- Parallel NetCDF (-mpi build only)
- CUDA (-cuda build only)
- FFTW

We therefore recommend installing some basic libraries *via*:

```
$ sudo apt-get install libblas-dev liblapack-dev libbz2-dev libnetcdf-dev
```

Should you encounter difficulties in the installation of CPPTRAJ, we refer to the official CPPTRAJ repository. All dependencies are open-source and freely available.

Usage

If you installed FEBISS and its dependencies via *febiss_setup*, you can use FEBISS to analyse trajectories for water placements, plot the data and select the waters you want to further investigate within a bar chart. To get a list of all available options and a useful input file, you can call

```
$ febiss_settings
```

This will place the file *all-settings.yaml* in your current directory. This input file requires only two alterations to be a valid input file. You must give the name of your topology file and the base name of your trajectory file(s). Along those two mandatory settings you find all other available settings both for the GIST analysis and the plotting of the retrieved data. Once you performed the analysis, you can also skip this step and directly plot the data. The analysis and plotting are done via calling the main program along with the yaml input file:

```
$ febiss all-settings.yaml
```

For a detailed description of all possible settings we refer to our full manual in our Github repository.

References

1. Weiss, A. K. H.; Hofer, T. S., Urea in aqueous solution studied by quantum mechanical charge field-molecular dynamics (QMCF-MD). *Molecular Biosystems* **2013,** *9* (7), 1864-1876.

2. Ishida, T.; Rossky, P. J.; Castner, E. W., A Theoretical investigation of the shape and hydration properties of aqueous urea: Evidence for nonplanar urea geometry. *J. Phys. Chem. B* **2004,** *108* (45), 17583-17590.

3. Jakalian, A.; Jack, D. B.; Bayly, C. I., Fast, efficient generation of high-quality atomic charges. AM1-BCC model: II. Parameterization and validation. *J. Comput. Chem.* **2002,** *23* (16), 1623-1641.

4. Bayly, C. I.; Cieplak, P.; Cornell, W. D.; Kollman, P. A., A Well-Behaved Electrostatic Potential Based Method Using Charge Restraints For Deriving Atomic Charges - The Resp Model. *J. Phys. Chem.* **1993,** *97* (40), 10269-10280.

5. Perdew, J. P., Density-Functional Approximation for the Correlation-Energy of The Inhomogeneous Electron-Gas. *Phys. Rev. B* **1986,** *33* (12), 8822-8824.

6. Becke, A. D., Density-Functional Exchange-Energy Approximation With Correct Asymptotic-Behavior. *Phys. Rev. A* **1988,** *38* (6), 3098-3100.
